# Macrophage DNases Limit Neutrophil Extracellular Trap–Mediated Defective Efferocytosis in Atherosclerosis

**DOI:** 10.1161/CIRCRESAHA.125.326353

**Published:** 2025-10-01

**Authors:** Umesh Kumar Dhawan, Tanwi Vartak, Hanna Englert, Stefan Russo, Luiz Ricardo C. Vasconcellos, Aarushi Singhal, Rahul Chakraborty, Karran Kiran Bhagat, Ciaran McDonnell, Mary Connolly, Edward Mulkern, Martin O’Donohoe, Mathias Gelderblom, Thomas Renne, Catherine Godson, Eoin Brennan, Manikandan Subramanian

**Affiliations:** William Harvey Research Institute, Queen Mary University of London, United Kingdom (U.K.D., S.R., A.S., K.K.B., M.S.).; Diabetes Complications Research Centre, Conway Institute and School of Medicine, University College Dublin, Ireland (T.V., C.G., E.B.).; Institute of Clinical Chemistry and Laboratory Medicine (H.E., T.R.), University Medical Center Hamburg-Eppendorf, Germany.; Department of Neurology (M.G.), University Medical Center Hamburg-Eppendorf, Germany.; The Francis Crick Institute, London, United Kingdom (L.R.C.V.).; CSIR-Institute of Genomics and Integrative Biology, New Delhi, India (R.C.).; Department of Vascular Surgery, Mater Misericordiae University Hospital, Dublin, Ireland (C.M., M.C., E.M., M.O.).; Irish Centre for Vascular Biology, School of Pharmacy and Biomolecular Sciences, Royal College of Surgeons in Ireland, Dublin, Ireland (T.R.).; Center for Thrombosis and Hemostasis (CTH), Johannes Gutenberg University Medical Center, Mainz, Germany (T.R.).

**Keywords:** atherosclerosis, efferocytosis, endoplasmic reticulum stress, extracellular traps, macrophages, neutrophils

## Abstract

**BACKGROUND:**

Neutrophil extracellular traps (NETs) contribute to atherosclerosis progression and are linked to adverse clinical outcomes such as myocardial infarction and stroke. Although the triggers of NET formation in plaques are known, the mechanisms governing DNase-mediated NET clearance and how these are disrupted during atherosclerosis remain unclear. Moreover, the consequences of impaired NET clearance on disease progression are not known.

**METHODS:**

Low-density lipoprotein receptor knockout (*Ldlr*^*−/*^^*−*^) mice with hematopoietic cell–specific deletion of DNase1 and DNase1L3 were fed a Western-type diet for 16 weeks to examine the impact of loss of DNase activity and the subsequent NET accumulation on advanced atherosclerosis. The effect of NETs on macrophage efferocytosis was examined in vitro and in the mouse peritoneal cavity and atherosclerotic plaque in vivo. To identify the signaling pathway impairing the NET-induced DNase response, in vitro assays were performed using selective endoplasmic reticulum stress pathway inhibitors, and the findings were validated in murine and human atherosclerotic tissues.

**RESULTS:**

Lack of DNase secretion by macrophages led to accumulation of NETs in local tissues, including atherosclerotic plaques. Persisting NETs in turn promoted cleavage of the efferocytosis receptor MerTK (c-mer proto-oncogene tyrosine kinase), resulting in defective macrophage efferocytosis and increased atherosclerotic plaque necrosis. In vitro screening identified endoplasmic reticulum stress–induced activation of the PERK (protein kinase R–like endoplasmic reticulum kinase)–ATF (activating transcription factor) 4 signaling axis in atherogenic macrophages as a key driver of impaired DNase secretion, leading to delayed NET clearance and their pathological persistence. Treatment of human atherosclerotic plaques and *Ldlr*^*−/−*^ mice with integrated stress response inhibitor, a selective PERK inhibitor, restored vascular DNase secretion and facilitated NET clearance.

**CONCLUSIONS:**

Macrophages play a key role in clearing NETs from tissues. Endoplasmic reticulum stress suppresses macrophage DNase secretion, leading to NET accumulation in atherosclerotic plaques, which triggers efferocytosis impairment and plaque progression. Targeting the PERK-ATF4 axis to restore DNase release and NET clearance represents a promising therapeutic strategy to promote plaque stabilization.

**Novelty and Significance**

**What Is Known?**

- Neutrophil extracellular traps (NETs) promote inflammation and tissue damage in several diseases, including atherosclerosis.
- DNases degrade NETs, but their cellular sources and regulatory mechanisms within atherosclerotic plaques are not well defined.
- NET accumulation worsens inflammation and destabilizes plaques, though the cellular mechanisms are not fully understood.

**What New Information Does This Article Contribute?**

- Macrophages are a major source of DNases that clear NETs in tissues, including atherosclerotic plaques.
- In plaque macrophages, endoplasmic reticulum stress activates the PERK (protein kinase R–like endoplasmic reticulum kinase)–ATF (activating transcription factor) 4 pathway, reducing DNase secretion and impairing NET clearance.
- Persistent NETs promote cleavage of the apoptotic cell recognition receptor MerTK, impairing efferocytosis and driving necrotic core expansion.
- Pharmacological PERK inhibition restores DNase secretion, enhances NET clearance, improves removal of dying cells, and reduces pathological plaque features in mice.

Efficient clearance of NETs is essential for resolving inflammation, but how this process is controlled within local tissues has remained unclear. This study identifies macrophages as a critical source of DNases that degrade tissue NETs. In atherosclerosis, endoplasmic reticulum stress activates PERK-ATF4 signaling in macrophages, impairing DNase secretion and NET clearance. As a result, NETs persist, driving cleavage of the apoptotic cell recognition receptor MerTK, which disables macrophages from clearing dying cells and promotes necrotic core expansion. Importantly, PERK inhibition restored DNase secretion, reduced NET burden, improved clearance of dying cells, and limited atherosclerosis progression in mice. These findings identify impaired macrophage DNase secretion as a previously unrecognized pathogenic mechanism sustaining NET persistence in atherosclerosis and highlight therapeutic strategies to restore resolution of inflammation by targeting endoplasmic reticulum stress pathways.

**Meet the First Author, see p 1229**

**Editorial, see p 1276**

Atherosclerotic cardiovascular disease remains a leading cause of morbidity and mortality worldwide.^1,2^ Although lipid-lowering therapies have significantly reduced cardiovascular disease–related deaths, a substantial residual risk for adverse clinical outcomes persists.^3^ Defective efferocytosis, the process of phagocytic clearance of apoptotic cells (ACs),^4^ and unresolved inflammation, are key drivers of the development of clinically dangerous necrotic atherosclerotic plaques.^5^ Therefore, novel strategies to promote efferocytosis and resolve inflammation are of interest.

Recently, neutrophil extracellular traps (NETs), web-like DNA structures released by neutrophils during NETosis, have emerged as a key driver of inflammation in atherosclerotic lesions and contribute to plaque progression.^6–8^ Several factors within the atherosclerotic plaque promote NETosis and release of NETs, including cholesterol crystals,^9^ CCL7 (chemokine [C-C motif] ligand 7),^8^ and lipid-induced oxidative stress.^10^ In addition, gene mutations associated with clonal hematopoiesis of indeterminate potential, including those encoding LNK (lymphocyte adaptor protein)^11^ and JAK2 (janus kinase 2),^12–14^ increase the susceptibility of neutrophils to release NETs, thereby exacerbating plaque progression and increasing cardiovascular disease mortality.

NETs are potent danger-associated molecular patterns that elicit a robust proinflammatory response.^15^ Therefore, the prompt degradation of NETs by extracellular endonucleases DNase1 and DNase1L3 is crucial for preventing tissue damage and resolving inflammation.^16,17^ We previously identified a physiological NET-induced DNase response pathway that operates in a feedback manner to maintain low NET levels in circulation. Moreover, we showed that NETs in systemic circulation trigger the release of DNases from the liver and intestine to rapidly degrade NETs and restore homeostasis.^18^ In contrast, the specific cells that express and release DNases in response to NETs generated within tissues, such as those in atherosclerotic plaques, are not characterized. Moreover, how the NET-induced DNase response is impaired on plaque progression, leading to accumulation of NETs is not understood. Although higher levels of lesional NETs correlate directly with increased plaque necrosis and clinical complications, whether NETs directly contribute to efferocytosis impairment and necrotic core formation is not known.

In this study, we show that lesional macrophages are critical for the generation of DNases and degradation of NETs in the atherosclerotic plaque. Moreover, using a combination of human and mouse atherosclerosis, we demonstrate that lipid-induced endoplasmic reticulum (ER) stress in atherogenic macrophages triggers activation of the PERK (protein kinase R–like endoplasmic reticulum kinase)–ATF (activating transcription factor) 4 signaling pathway to impair the NET-induced DNase response with consequent accumulation of NETs in advanced atherosclerosis. Finally, we demonstrate that NETs directly impair macrophage efferocytosis, thereby contributing to necrotic core expansion and the formation of vulnerable plaques.

## Methods

### Data Availability

Data supporting the findings in this study are available from the corresponding author on reasonable request. A list of major resources used is available in Table S1.

### Animals and Animal Maintenance

C57BL/6J mice were purchased from Charles River (United Kingdom). Low-density lipoprotein receptor knockout *(Ldlr*^*−/−*^) mice (stock no. 002207) were purchased from The Jackson Laboratory. *Dnase1/Dnase1l3*^*−/−*^ mice^17^ were bred and maintained in-house. The mice were housed in individually ventilated cages at the Biological Service Unit at Queen Mary University of London under specific pathogen-free conditions with a 12-hour light/dark cycle and had unrestricted access to food and water. All animal experimental procedures performed were approved by the Home Office, United Kingdom. Bones from MerTK cleavage–resistant (MerTK^CR^) knockin mice^19^ were a kind gift from Prof Ira Tabas, Columbia University Medical Center, NY. *Tlr4*^*−/−*^ mice^20^ were obtained from The Francis Crick Institute under an Material Transfer Agreement from Osaka University. For the induction of atherosclerosis, *Ldlr*^*−/−*^ mice were fed a Western-type diet (46% Kcal from fat and 0.2% cholesterol, from sodium dodecyl sulfate, catalog no. 829100) for 16 weeks.

### Neutrophil Differentiation and Isolation of NETs

Human HL-60 (acute promyelocytic leukemia) cells were purchased from American Type Culture Collection (ATCC) and cultured in RPMI-1640 media supplemented with 1 mmol/L sodium pyruvate, 10% (vol/vol) fetal bovine serum, and 100 U/mL penicillin-streptomycin. HL-60 cells were seeded at 10 million cells per T-75 flask and treated with 1 µmol/L All-trans retinoic acid for 4 days to differentiate into neutrophil-like cells. For induction of NETosis, bone marrow–derived neutrophils and differentiated HL-60 cells were resuspended with serum-free medium and subsequently treated with phorbol 12-myristate 13-acetate (100 nmol/L), or 7-ketocholesterol (50 µg), for 4 hours. Adherent NETs were scraped from the surface and collected, together with cells and the culture supernatant. Cells and cellular debris were separated from the supernatant using low-speed centrifugation at 450*g* for 10 minutes at 4 °C. Subsequently, the supernatant enriched with NETs was subjected to high-speed centrifugation at 18 000*g* for 30 minutes at 4 °C. The pelleted NETs were then resuspended in ice-cold PBS and stored at −80 °C until further use.

### Mouse Bone Marrow–Derived Macrophages

Eight- to 12-week-old female mice were euthanized, and their femur and tibia were collected. Bones were flushed with media and filtered through a 100 µm filter, followed by centrifugation at 300*g* for 5 minutes. Red blood cells were lysed by incubation with red blood cell lysis buffer (R7757; Sigma) for 2 minutes, followed by pelleting. Cells were resuspended and cultured in DMEM high glucose supplemented with 10% FBS, penicillin-streptomycin, and 20% L-929 cell culture supernatant.^21^ Half the medium was replaced on day 3, and cells were cultured until day 7 to achieve complete macrophage differentiation.

For macrophage polarization, bone marrow–derived macrophages were exposed to LPS (lipolpolysaccharide, 20 ng/mL)+IFN (interferon) γ (10 ng/mL) for M1-phenotype or to IL (interleukin)-4 (20 ng/mL) for 24 hours for M2 polarization.

### Aortic Explant Culture Protocol

Mice were euthanized, and after intraventricular perfusion with PBS, the entire aorta—from the carotid artery to the iliac artery bifurcation—was dissected under a dissection microscope. The aorta was carefully cleaned to remove any excess fat from its outer layer. It was then sectioned into 1 mm fragments using a fine scalpel blade, and the fragments were randomized. The aortic explants were cultured for 4 hours in RPMI-1640 medium supplemented with 1 mmol/L sodium pyruvate, 10% (vol/vol) fetal bovine serum, and 100 U/mL penicillin-streptomycin. After 4 hours, the medium was replaced with serum-free medium, and the explants were treated with or without NETs (500 ng/mL). The spent medium was collected, and residual NETs in the culture supernatant were quantified using the Picogreen assay. The supernatant was concentrated 100× using a 10 kDa filter, followed by measurement of DNase activity by single radial enzyme diffusion. For specific experiments, the explants were either left untreated or pretreated overnight with the ER stress relievers tauroursodeoxycholic acid (TUDCA; 50 μg/mL) or integrated stress response inhibitor (ISRIB; 0.5 μM) before exposure to NETs. Human carotid endarterectomy specimens from patients with symptomatic atherosclerosis were obtained after informed consent and approval of the ethics committee of Mater Misericordiae University Hospital (1/378/2372). Human plaque processing and dissection were performed as previously described.^22^ Human carotid endarterectomy tissues were used for DNase assay and NET clearance as described above.

### Single Radial Enzyme Diffusion Assay

DNase buffer (20 mmol/L Tris-HCl, pH 7.8, 10 mmol/L MnCl2, 2 mmol/L CaCl_2_) was prepared and mixed with salmon testes DNA (55 µg/mL) and 2× SYBR Safe. The mixture was incubated at 50 °C for 10 minutes, followed by the addition of an equal volume of 2% agarose dissolved in nuclease-free water. The resulting solution was poured into plastic trays and allowed to solidify at room temperature. Wells ≈0.1 mm in size were created in the gel using a 20 µL pipette tip. Subsequently, 2 µL of plasma, 5 µL of 100× concentrated cell culture supernatant and peritoneal fluid, or 5 µL of tissue extract were loaded into the wells. Tissue extracts for DNase activity were prepared by homogenizing tissues using bead homogenization in Tris-HCl, pH 7.8. The crude extracts were centrifuged at 20 000 *g* for 10 minutes at 4 °C, and the supernatants were collected. The gels were incubated for 6 hours at 37 °C in a humidified chamber. After incubation, images of the gels were captured using the Azure 400 gel documentation system. DNA degradation was quantified using Fiji software by comparing the samples against a standard curve generated from known concentrations of purified DNase1 run on the same gel.

### Denaturing Polyacrylamide Gel Electrophoresis Zymography

To measure individual DNase1 and DNase1L3 activity, 10% (v/v) resolving gels were made by mixing with DNA extracted from salmon testes (200 μg/mL). For DNase1 activity assessment, 0.5 µL and 5 µL of 100× concentrated cell culture supernatant and peritoneal exudate were mixed with 12 µL of nuclease-free water and 5 µL of sodium dodecyl sulfate gel-loading buffer. The solution was heated for 5 minutes and then loaded onto the gels. After electrophoresis, gels were washed twice with 10 mmol/L Tris/HCl pH 7.8, at 50 °C for 30 minutes to remove remnant sodium dodecyl sulfate. Gels were transferred into refolding buffer (10 mmol/L Tris/HCl pH 7.8, 3 mmol/L CaCl2, 3 mmol/L MgCl_2_) and incubated at 37 °C overnight.

For DNase1L3 detection, 2 µL of serum was used, and samples were mixed with 1 µL of beta-mercaptoethanol before loading onto the gel. Electrophoresis and subsequent washing steps were carried out as described above, and the gel was incubated for 48 hours at 37 °C in refolding buffer 1 (10 mmol/L Tris/HCl pH 7.8, 1 mmol/L beta-mercaptoethanol), followed by another 48-hour incubation at 37 °C in refolding buffer-2 (refolding buffer 1 supplemented with 3 mmol/L CaCl_2_ and 3 mmol/L MnCl_2_). Images were captured using a Gel Doc system (FluorChem E), and quantification was conducted using Fiji software.

### Quantification of NETs by Myeloperoxidase-DNA ELISA

To quantify NETs, polyclonal anti-MPO (myeloperoxidase) antibodies were coated onto 96-well ELISA plates using a carbonate-bicarbonate buffer at a dilution of 1:1000 for 12 hours at 4 °C. After a 1-hour blocking step at room temperature with 3% bovine serum albumin (BSA), the wells were incubated for 12 hours at 4 °C with 50 µL of plasma, 200 µL of peritoneal exudate, and 200 µL of cell culture supernatant. After 3 washes with PBS containing 0.1% Tween-20, the DNA content was quantified using the Quant-iT PicoGreen dsDNA assay kit, following the manufacturer’s instructions. For positive and negative controls, appropriate wells were incubated with in vitro–generated NETs and genomic DNA, respectively.

### Soluble MerTK ELISA

Soluble MerTK levels were measured using a Mouse Mer DuoSet ELISA kit (R&D Systems) following the manufacturer’s instructions. ELISA plates were coated with a capture antibody overnight, blocked with 1% BSA, and incubated with samples or standards for 2 hours at room temperature. After adding the detection antibody conjugated to HRP (horseradish peroxidase)-streptavidin, the TMB (3,3′,5,5′-tetramethylbenzidine) substrate was applied, and the reaction was stopped. Absorbance was measured at 450 nm.

### In Vitro Efferocytosis Assay

HL-60 cells were labeled with CellVue Claret according to the manufacturer’s protocol. Briefly, cells were washed with PBS and then resuspended in 2 mL of Diluent C along with 4 µL of the dye. CellVue Claret-labeled cells were exposed to ultraviolet-C (UV-C) radiation (254 nm) for 7 minutes, equivalent to 5 kJ/m^2^, followed by a 1-hour incubation at 37 °C to induce apoptosis.

Bone marrow–derived macrophages were plated at a density of 0.5 million cells per well in a 12-well plate. Apoptotic HL-60 cells were added at a ratio of 1:1 ACs to macrophages and incubated for 1 hour. After the incubation period, unengulfed ACs were washed away, and images were captured using a fluorescence microscope (EVOS FL) at ×20 magnification. FIJI (ImageJ) was used to quantify efferocytosis efficiency by calculating the percentage of macrophages that have engulfed an AC.

### Quantification of Efficiency of NET Clearance

For the NET clearance assay, bone marrow–derived macrophages were incubated with NETs (250 ng/mL) in serum-free media. At the specified time points for each experiment, the amount of remaining NETs in the supernatant was quantified using the Quant-iT PicoGreen dsDNA assay kit, following the manufacturer’s protocol. The percentage of NETs remaining was calculated relative to the initial amount of NETs added.

### RNA Isolation, cDNA Preparation, and Quantitative Polymerase Chain Reaction

Total RNA was extracted from cells using the RNeasy Mini Kit according to the manufacturer’s instructions. For total RNA extraction from the aorta, the heart and entire aorta were perfused, placed on a silicone plate, and pinned. Excess fat was removed, and the aortic arch was isolated and transferred into Precellys tubes containing 500 µL of Trizol. The tissue was homogenized using a Precellys 24 Tissue Homogenizer at 6500 rotations per minute (rpm) for 30 seconds across 20 cycles. Total RNA was then isolated using the chloroform-isopropanol method.^23^ For mRNA expression analysis, 1 µg of total RNA was reverse-transcribed into cDNA using the PrimeScript first-strand cDNA synthesis kit (TaKaRa). The cDNA was mixed with KAPA SYBR Green master mix, and quantitative polymerase chain reaction was performed using the Roche Lightcycler 480. The results were obtained as cycle threshold values, which were used to calculate 2-ΔΔ cycle threshold and determine fold changes, providing relative mRNA expression levels normalized to an endogenous housekeeping gene.

### Bone Marrow Transplantation

Ten-week-old *C57BL/6* (female or male) or female *Ldlr*^*−/−*^ recipient mice underwent lethal X-ray irradiation, administered in 2 equal doses of 4 Gy, separated by a 4-hour interval. After the second radiation dose, 5 million donor bone marrow cells were delivered via tail vein injection. After a 6-week period for bone marrow reconstitution, the mice were used for subsequent experiments.

### In Vivo DNase Response and Efferocytosis in the Peritoneal Cavity

To quantify the NET-induced DNase response in the peritoneal cavity, appropriate groups of 10-week-old female C57BL/6J mice were administered either NETs (1 mg) or a vehicle control (PBS) intraperitoneally. Three hours later, the mice were euthanized, and peritoneal lavage was performed with 5 mL of cold PBS. The peritoneal lavage fluid was centrifuged at 18 000*g* for 10 minutes and concentrated 100× for analysis of DNase activity using single radial enzyme diffusion and denaturing polyacrylamide gel electrophoresis zymography.

For analysis of efferocytosis efficiency, appropriate groups of mice were injected with 10^6^ CellVue Claret-labeled ACs in 300 μL of PBS into the peritoneal cavity of mice that were previously exposed to NETs or vehicle for 2 hours. One hour after injection of the ACs, the mice were euthanized, and peritoneal lavage was performed. The cells in the peritoneal lavage were immunostained with fluorescently labeled anti–F4/80 antibody. Efferocytosis was analyzed by identifying the F4 to 80+ CellVue Claret+ population by flow cytometry.

### Adoptive Transfer of Macrophages Into the Peritoneal Cavity

Age-matched female wild-type (WT) and *Dnase1/Dnase1l3*^*−/−*^ mice resident peritoneal macrophages were isolated using the macrophage enrichment kit (Miltenyi Biotech), and 0.5×10^6^ macrophages were injected intraperitoneally into the recipient female C57BL/6J mice. 16 hours postadoptive cellular transfer, the mice were injected with NETs and ACs as described above to quantify the DNase response and macrophage efferocytosis efficiency.

### Atherosclerotic Plaque Morphometry, Immunofluorescence, and In Situ Efferocytosis Assay

At the end of the experiment, the mice were euthanized, blood collected, and tissues perfused with 1× PBS via intraventricular injection. The heart, along with the intact aortic root, was fixed in 10% formalin, embedded in paraffin, and sectioned into 8 µm slices using a microtome. Fifty consecutive sections were obtained from the initial appearance of the aortic valve. Hematoxylin and eosin staining of 6 equally spaced sections per mouse covering the entire aortic root was analyzed for quantification of total lesion area and necrotic area. In addition, sections were stained with Mason’s trichrome dye (Sigma-Aldrich) following the manufacturer’s protocol to quantify lesional collagen content.

For immunostaining of formalin-fixed paraffin-embedded specimens, deparaffinization was conducted using Histoclear, followed by rehydration with graded ethanol and final rinses with 1× PBS. Antigen retrieval was achieved by incubating sections in sodium citrate buffer (10 mmol/L Sodium citrate, 0.05% Tween-20, pH 6.0) at 95 °C for 45 minutes. After blocking with 3% FBS in 1× PBS for 30 minutes, the sections were incubated overnight at 4 °C with appropriate primary antibody. After washes, the sections were incubated with appropriate fluorescently labeled secondary antibody for 1 hour at room temperature and counterstained with DAPI (4′,6-diamidino-2-phenylindole).

For in situ efferocytosis assay, terminal deoxynucleotidyl transferase dUTP nick-end labeling (TUNEL) staining was performed according to the manufacturer’s instructions. After TUNEL labeling, the sections were immunostained with anti-Mac2 (galectin-3) antibody as described above and counterstained with DAPI. Images were captured using a fluorescence microscope (EVOS FL), and the in situ efferocytosis efficiency was determined as the ratio of TUNEL+ACs that are associated with a Mac2+ macrophage (associated) or lying free (associated:free). The analysis of all atherosclerotic lesions was conducted in a blinded manner, with one individual executing the experiment and another person performing the analysis.

### Silencing RNA (siRNA) Transfection

Lipofectamine RNAimax transfection reagent was used for siRNA transfection following the manufacturer’s protocol. Briefly, 2×10^6^ HL-60 cells were seeded in 6-well plates and treated with ATRA (all-trans retinoic acid) for 4 days before proceeding with knockdown. Similarly, 0.5×10^6^ bone marrow–derived macrophages were seeded in 12-well plates and incubated overnight. To generate siRNA-lipofectamine complexes, Lipofectamine RNAimax reagent was diluted in Opti-MEM and combined with 10 pmol of siRNA targeting ATF4 or HMGB1 (high-mobility group box 1) in an equal volume of Opti-MEM. This mixture was incubated for 5 minutes at room temperature and then added to the cells. After 12-hour culture in Opti-MEM, cells were transferred to DMEM supplemented with 10% (vol/vol) FBS and 100 U/mL penicillin-streptomycin for 24 hours before proceeding with further experiments.

### Immunofluorescence Staining of NETs

Twenty-four-well plates were precoated with poly l-lysine for 1 hour before seeding with 0.5×10^6^ ATRA-differentiated HL-60 cells, followed by incubation overnight. The culture media was replaced with fresh serum-free media and treated with 100 nmol/L phorbol 12-myristate 13-acetate for 4 hours, followed by fixation with 4% paraformaldehyde.

For immunostaining of NETs in the peritoneal exudate, 500 µL of the exudate was incubated with poly l-lysine–coated dishes at 4 °C. Samples were then blocked with 3% FBS in 1×-PBS, followed by incubation with primary antibody against citrullinated histone H3, MPO, or HMGB1 and subsequent incubation with fluorophore-conjugated secondary antibody. Nuclei were counterstained using DAPI. Images were captured using a fluorescence microscope and analyzed with FIJI software.

### Western Blotting

Cells were lysed in 2× Laemmli buffer with 1% v/v dithiothreitol and heated at 95 °C for 10 minutes. Samples were then either stored at −20 °C or used immediately. Samples were loaded onto a 10% polyacrylamide gel along with a prestained ladder, and electrophoresis was performed. Proteins were transferred to a polyvinylidene fluoride Membrane via wet transfer at 90 volts for 90 minutes. The membrane was blocked with 5% milk or 5% BSA for 1 hour at room temperature, followed by a brief washing. Primary antibodies were then incubated overnight at 4 °C, and after washes, the membrane was incubated with the appropriate secondary HRP-conjugated antibody for 1 hour at room temperature. After further washing, 1 mL of Western blotting HRP substrate was used for chemiluminescent imaging using an Azure 400 gel documentation system.

### Mass-Spectrometric Analysis

NETs were digested by using sequencing-grade trypsin (1:10 ratio, trypsin:protein) for 16 to 18 hours at 37 °C. Before digestion, the sample was reduced with 25 mmol/L dithiothreitol for 30 minutes at 56 °C, and the cysteines were blocked by 55 mmol/L IAA at room temperature for 15 to 20 minutes. Digested peptides were reconstituted in 5 µL of LC-MS grade water containing 0.1% formic acid and run on a quadrupole-time of flight (TOF) hybrid mass spectrometer (TripleTOF6600; Sciex) coupled to a nano-LC system (Eksigent NanoLC-400). Peptides were loaded onto a reverse phase peptide ChromoLC trap (200 mm 0.5 mm) column and separated using a C18 column (75 mm 15 cm; Eksigent). The samples were run using buffers A (99.9% Liquid Chromatography-Mass Spectrometry water+0.1% formic acid) and B (99.9% acetonitrile+0.1% formic acid) as the mobile phases. At a constant flow rate of 250 nL min^−^1, the gradient is composed of 95% buffer A for 2 minutes, 90% buffer A for 8 minutes, 20% buffer A for 42 minutes, and then 95% buffer A for 16 minutes. Data were acquired with a Nano Spray source installed in the TripleTOF 6600 System using a nebulizing gas of 20 psi, a curtain gas of 25 psi, an ion spray voltage of 2000 V, and a heater interface temperature of 75 °C. Data-dependent acquisition mode was set up with a TOF/ms survey scan (350–1600 m/z) with an accumulation time of 250 ms. A maximum of 10 precursor ions per cycle were chosen for fragmentation, with each tandem mass spectrometry (MS/MS) spectrum (200–1800 m/z) being accumulated for 70 ms, for a total cycle time of roughly 2.05 seconds. Parent ions were chosen for MS/ms fragmentation if their abundance was >150 cps and their charge state ranged from +2 to +5. The protein pilot v5.0 was used to analyze the.wiff files produced by the TripleTOF 6600, which include both MS and MS/ms spectra, to identify the desired protein.

### Statistical Analysis

All data are presented as mean±SEM. The number of biological replicates or the number of mice used in each experiment is indicated in the figure legends. Data that passed the Shapiro-Wilk test for normal distribution were analyzed using a parametric test. Nonparametric tests were used where the sample size was <10 or where the data did not show normal distribution. The specific test used for each analysis is indicated in the figure legends. All statistical analyses were performed using GraphPad Prism Version 10.0. The figure shows *P* values for comparisons that reached statistical significance (*P*<0.05).

## Results

**Figure 1.**
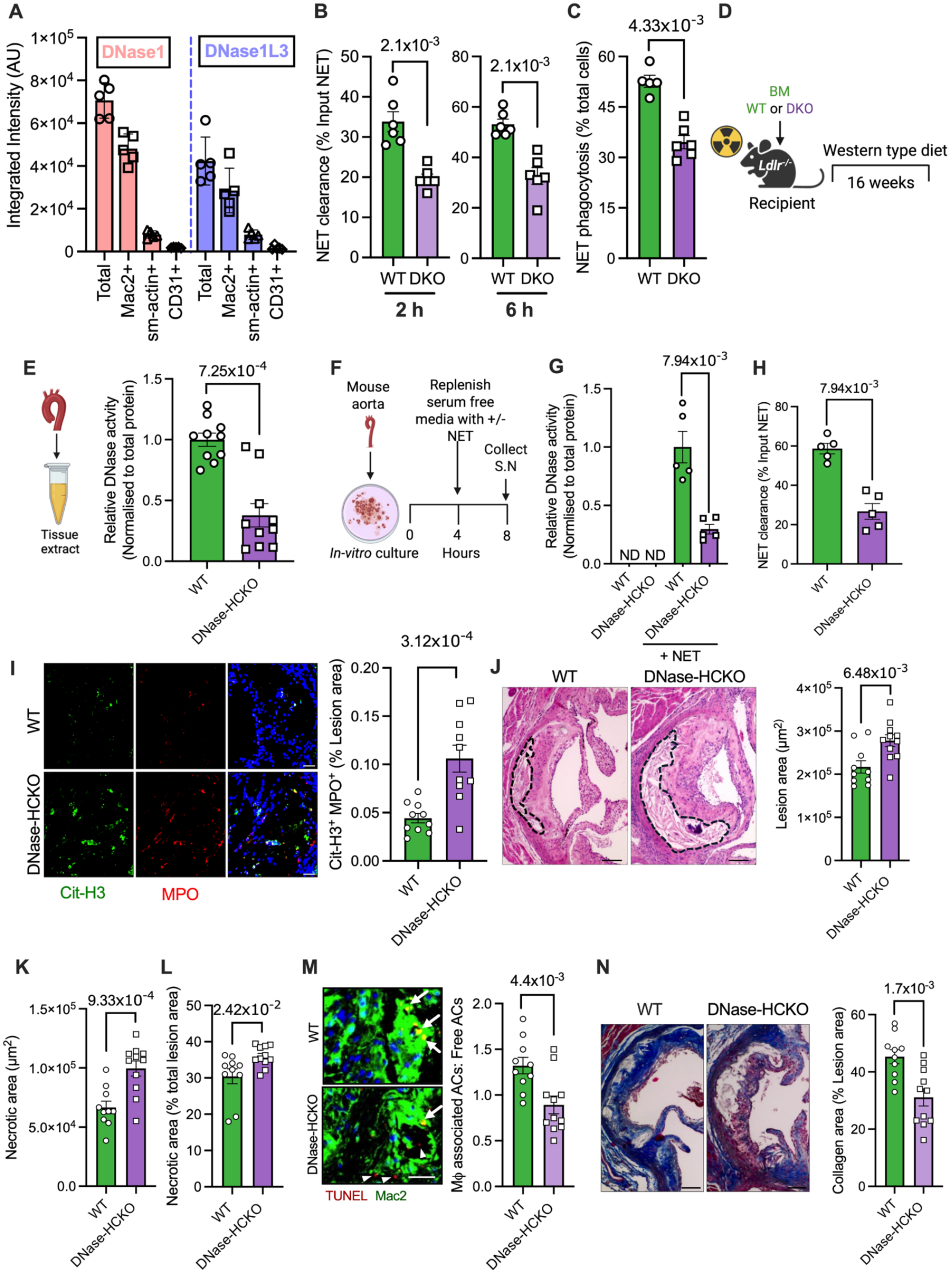
**DNase deficiency in hematopoietic cells impairs neutrophil extracellular trap (NET) clearance in atherosclerotic plaques. A**, The relative levels of DNase1 and DNase1L3 in lesional macrophages (Mac2+), smooth muscle cells (sm-actin [smooth muscle actin]+), and endothelial cells (CD31+) were quantified by measuring DNase1 and DNase1L3 fluorescence intensity in antibody-labeled aortic root sections of 16-week Western diet–fed *Ldlr*^*−/−*^ mice. **B**, Bone marrow–derived macrophages (BMDMs) from wild-type (WT) and *Dnase1*^*−/−*^*Dnase1l3*^*−/−*^ (double knockout [DKO]) mice were exposed to NETs (250 ng/mL) for either 2 or 6 hours. The quantity of remaining NETs in the supernatant was measured by Picogreen assay, and the NET clearance efficiency was determined relative to input NET concentration. **C**, BMDMs from WT and DKO mice were incubated for 2 hours with pHrodo-red labeled NETs. After washes, fluorescence microscopy was performed, and the percentage of macrophages that showed engulfment of fluorescent NETs was quantified. **D**, Eight-week-old female *Ldlr*^*−/−*^ mice were lethally irradiated followed by administration of bone marrow cells from either WT or DKO mice. Six weeks postbone marrow reconstitution, WT and hematopoietic cell–specific DNase1/DNase1L3 knockout (DNase-HCKO) mice were fed a Western-type diet for 16 weeks and then euthanized for analysis. **E**, Brachiocephalic artery from WT and DNase-HCKO mice were homogenized, and the tissue extract was analyzed for DNase activity by single radial enzyme diffusion assay. n=10 mice per group. **F**, Aortic tree from both groups of mice (n=5) were harvested, fragmented, and maintained in culture as explants, followed by exposure to vehicle or NETs for 4 hours. The supernatant was collected for measurement of (**G**) DNase activity and (**H**) NET clearance efficiency. **I**, Lesional NET level was quantified by immunostaining for MPO (myeloperoxidase) and citrullinated histone H3 (Cit-H3) in DAPI (4′,6-diamidino-2-phenylindole)–stained aortic root sections of WT and DNase-HCKO mice. n=10 mice per group. Scale bar, 50 µm. **J**, Hematoxylin and eosin–stained aortic root sections were analyzed for total atherosclerotic lesion area, (**K**) necrotic area, and (**L**) plaque necrosis as a percentage of total lesion area. The regions of plaque necrosis are demarcated by the black dashed line. n=10 to 11 mice per group. Scale bar, 50 µm. **M**, In situ efferocytosis assay in aortic root sections labeled with terminal deoxynucleotidyl transferase dUTP nick-end labeling (TUNEL) reagent to detect apoptotic cells (ACs; red) followed by Mac2 immunolabelling (green) to identify lesional macrophages. Lesional efferocytosis efficiency was calculated as the ratio of macrophage-associated ACs to free-lying ACs. White arrows indicate ACs associated with macrophages, whereas white arrowheads show free-lying TUNEL+ cells. n=10 mice per group. Scale bar, 25 µm. **N**, Quantification of lesional collagen content in Mason’s trichrome-stained aortic root sections. n=10 mice per group. Scale bar, 50 µm. All data are represented as mean±SEM. Test for normality was conducted by the Shapiro-Wilk test. *P* value was determined by the Mann-Whitney *U* test (**B**, **C**, **E**, **G**, and **H**), or unpaired *t* test (**I** through **N**). AU indicates arbitrary units; Mφ, macrophage; ND, not detected; and S.N., supernatant.

To characterize the lesional cells that express DNase1 and DNase1L3 and therefore could participate in clearance of NETs via secretion of DNases, we performed immunostainings for DNase1 and DNase1L3 along with cell type–specific markers for major lesional cell types, including smooth muscle cells, macrophages, and endothelial cells. Murine atherosclerotic plaques showed expression of DNase1 and DNase1L3 in all lesional cell types examined (Figure S1A). However, immunofluorescence signal identified macrophages as the major source of plaque DNase (Figure 1A). Indeed, both human and mouse macrophages release DNase1 and DNase1L3 in response to NETs.^18^ Because the role of macrophage DNases in the clearance of NETs is not known, we compared the NET clearance efficiency of *Dnase1*^*−/−*^*Dnase1l3*^*−/−*^ (double knockout [DKO]) macrophages with WT macrophages. We observed that deficiency of DNase1 and DNase1L3 in macrophages impairs their ability to degrade NETs (Figure 1B) as well as their subsequent engulfment by macrophages (Figure 1C). Based on these data, we hypothesized that macrophages are critical for the clearance of NETs in atherosclerotic plaques via the release of DNases.

To test this hypothesis, we generated bone marrow chimeric mice that are deficient in DNase1 and DNase1L3 in all cells of hematopoietic origin by adoptively transferring bone marrow cells obtained from *Dnase1*^*−/−*^*Dnase1l3*^*−/−*^ mice into lethally irradiated atherosclerosis-prone *Ldlr*^*−/−*^ mice (hematopoietic cell–specific DNase1/DNase1L3 knockout [DNase-HCKO]). The control group was injected with bone marrow cells obtained from WT mice. Six weeks after bone marrow transplantation, both groups of mice were fed a Western-type diet for 16 weeks to generate advanced atherosclerosis (Figure 1D). Aortic extracts were analyzed for DNase activity by single radial enzyme diffusion assay. Interestingly, we observed that the basal vascular DNase activity was lower in the DNase-HCKO mice as compared with WT mice (Figure 1E), suggesting that hematopoietic cell–derived DNase is critical for maintenance of basal tissue DNase activity. Important to note that plasma level of NETs (Figure S1B) as well as plasma DNase activity was unaffected in the chimeric mice (Figure S1C), demonstrating that hematopoietic cell–derived DNases do not contribute significantly towards the maintenance of circulatory DNase activity.

Next, to address the question of relative contribution of hematopoietic versus Nonhematopoietic cells in the release of DNase in response to NETs within the atherosclerotic artery, we developed an ex vivo assay wherein atherosclerotic vascular tissue explants obtained from 16-week Western diet–fed WT or DNase-HCKO mice were exposed to either NETs or vehicle and the extent of release of DNase in the supernatant was quantified (Figure 1F). In the absence of exogenously added NETs, the explant tissue did not release detectable levels of DNase (Figure 1G). However, when NETs were added to the explant culture, DNase was released into the supernatant, demonstrating the presence of an active NET-induced DNase response in the vascular explant tissue (Figure 1G). Interestingly, compared with the WT mice, there was ≈70% decrease in DNase activity in the supernatant of aortic tissue obtained from the DNase-HCKO mice (Figure 1G). More importantly, this decrease in DNase activity was associated with an impairment in the clearance of exogenously added NETs (Figure 1H).

Based on these data, we questioned whether the decrease in plaque DNase activity in the DNase-HCKO mice is associated with increased accumulation of NETs. Indeed, DNase-HCKO mice showed increased signal of lesional citrullinated histone H3^+^ MPO-DNA complexes in immunofluorescence, indicative of increased accumulation of NETs (Figure 1I). The colocalization of the citrullinated histone H3^+^MPO^+^ signal with the neutrophil marker Ly6G, but not the macrophage marker Mac2 (Figure S1D), suggests that most of these extracellular traps are neutrophil derived. This accumulation is primarily due to defective DNase-mediated NET clearance, rather than increased NET formation, as there was no statistically significant difference between WT and DNase-HCKO mice in peripheral blood neutrophil numbers, neutrophil activation profiles, and in the induction of NETosis on exposure to phorbol 12-myristate 13-acetate (Figure S1E through S1G). From a disease perspective, the impaired clearance of NETs in the DNase-HCKO mice was associated with an increase in atherosclerotic plaque area (Figure 1J). The increase in lesion area was not due to metabolic perturbations because body weight, plasma cholesterol, triglycerides, and blood glucose showed no statistically significant difference between the WT and DNase-HCKO mice (Figure S1H through S1K). More importantly, plaque necrosis, which is a critical determinant of vulnerable plaque formation, was increased in the DNase-HCKO mice (Figure 1K). This increase was not due to larger lesions in the DNase-HCKO mice because the percentage lesion area that is necrotic was also higher (Figure 1L). Because plaque necrosis is driven primarily by a defective macrophage efferocytosis,^24^ we tested whether lesional efferocytosis efficiency is impaired in the DNase-HCKO mice using an in situ efferocytosis assay.^25^ Accordingly, aortic root sections were labeled with TUNEL reagent to detect intimal ACs and then costained with Mac2 to detect macrophages. The ratio of TUNEL+ nuclei that are associated with a macrophage to those that are lying free was used as an indicator of the in situ efferocytosis efficiency.^26^ Consistent with the plaque necrosis data, the DNase-HCKO mice demonstrated significant impairment in macrophage efferocytosis efficiency (Figure 1M). Notably, the total number of lesional ACs showed no statistically significant difference between the 2 groups (Figure S1L), suggesting that the increased plaque necrosis is driven by defective efferocytosis. Moreover, the DNase-HCKO mice showed decreased lesional collagen content (Figure 1N). Taken together, these data demonstrate that DNase-HCKO mice have an accelerated atherosclerotic plaque progression with characteristics of plaque instability, such as decreased collagen and larger necrotic area, which in humans is associated with adverse clinical outcomes such as myocardial infarction and stroke.

### DNase Deficiency Leads to NET Accumulation and Impairment of Macrophage Efferocytosis

**Figure 2.**
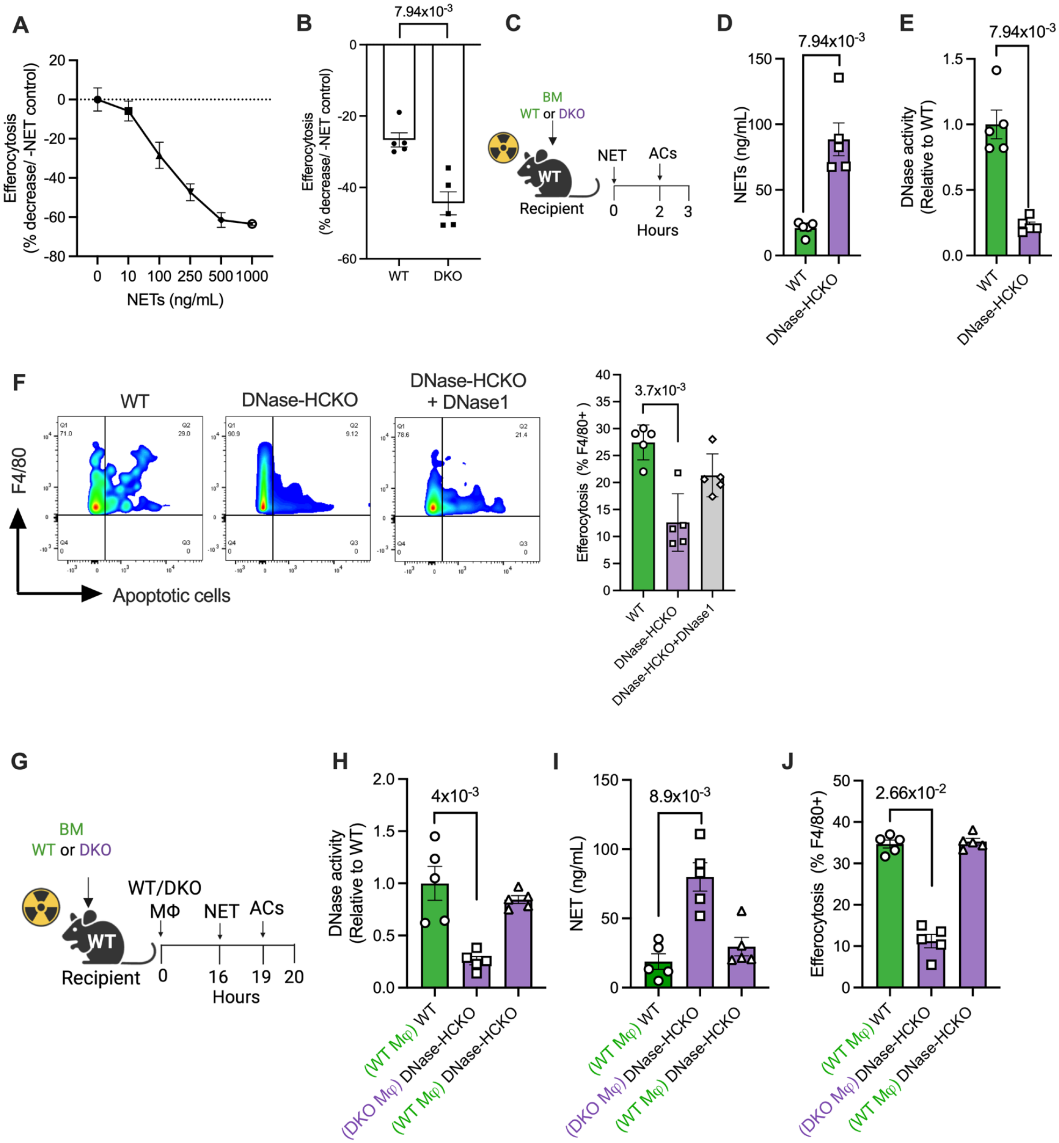
**Impaired neutrophil extracellular trap (NET) clearance promotes defective macrophage efferocytosis. A**, Bone marrow–derived macrophages (BMDMs) were treated with vehicle or NETs at indicated concentrations for 2 hours, followed by incubation with fluorescently labeled apoptotic cells (ACs) for 1 hour. Efferocytosis efficiency was quantified by fluorescence microscopy and expressed as a percentage decrease relative to the vehicle group. n=4 biological replicates. **B**, Wild-type (WT) and double knockout (DKO) BMDMs were exposed to vehicle or NETs (250 ng/mL) for 2 hours, then incubated with ACs for 1 hour. Efferocytosis efficiency is shown as a percentage change relative to the vehicle group. n=5 biological replicates. **C**, Eight-week-old female C57BL/6J mice were lethally irradiated, followed by injection of bone marrow cells from either WT or DKO mice. Six weeks posttransplant, WT and hematopoietic cell–specific DNase1/DNase1L3 knockout (DNase-HCKO) mice were injected intraperitoneally with NETs. A subgroup of DNase-HCKO mice also received purified DNase1 with NETs. Two hours later, CellVue Claret-labeled ACs were injected intraperitoneally. Mice were euthanized 1 hour later, and peritoneal lavage was analyzed for (**D**) DNase activity by single radial enzyme diffusion, (**E**) NET levels by picogreen assay, and (**F**) macrophage efferocytosis efficiency by flow cytometry. n=5 mice per group. **G**, WT, and DNase-HCKO chimeric mice were generated as above. WT mice received adoptive transfer of WT macrophages, whereas DNase-HCKO mice received WT or DKO macrophages. Sixteen hours after adoptive cell transfer, the mice were injected with NETs followed by ACs as described above for the measurement of peritoneal lavage (**H**) DNase activity, (**I**) NET levels, and (**J**) efferocytosis efficiency. n=5 mice per group. The data are represented as mean±SEM. *P* values were calculated using Mann-Whitney *U* test (**B**, **D**, and **E**) and Kruskal-Wallis test with Dunn multiple comparisons correction (**F**, **H** through **J**). Mφ indicates macrophage.

Because the accumulation of NETs in the DNase-HCKO mice was associated with decreased lesional efferocytosis efficiency, we asked whether NETs directly impair macrophage efferocytosis. Indeed, when macrophages were incubated with NETs in vitro, there was a concentration-dependent decrease in the uptake of ACs (Figure 2A), suggesting that NETs decrease the efficiency of AC clearance. Consistently, the NET-induced decrease in efferocytosis was more pronounced in the *Dnase1*^*−/−*^*Dnase1l3*^*−/−*^ (DKO) macrophages (Figure 2B; Figure S2A), which have impaired NET clearance (Figure 1B) and therefore persistently higher concentrations of NETs. WT and DKO macrophages show no statistically significant difference in efferocytosis efficiency in the absence of NETs (Figure S2B), demonstrating that the DKO macrophages do not have an intrinsic defect in efferocytosis. Taken together, these data suggest that a decrease or loss of NET-induced DNase secretion by macrophages leads to decrease in DNase-mediated clearance and accumulation of NETs, which impairs macrophage efferocytosis.

Next, to address the question of whether accumulation of NETs impairs macrophage efferocytosis in vivo, we injected NETs into the peritoneal cavity of bone marrow chimeric C57BL/6 mice that were reconstituted with either WT or DKO bone marrow cells. Two hours later, the mice were given an intraperitoneal injection of fluorescently labeled ACs. One hour after AC injection, peritoneal lavage was performed (Figure 2C). As with the atherosclerotic mice, the DNase-HCKO mice had decreased levels of DNase in the peritoneal lavage as compared with the WT mice (Figure 2D). Most importantly, the decreased DNase activity in the DNase-HCKO mouse was associated with higher levels of uncleared NETs (Figure 2E) and decreased macrophage efferocytosis (Figure 2F), which could be rescued by administration of intraperitoneal DNase1 (Figure 2F). These data suggest that loss of NET-induced production of DNases by hematopoietic cells in tissues leads to delayed clearance and accumulation of NETs with consequent impairment in macrophage efferocytosis.

Finally, to directly test whether macrophages are the major cellular source of the NET-induced DNase response in tissues, we adoptively transferred equal numbers of either WT or DNase-deficient macrophages into the peritoneal cavity of DNase-HCKO mice (Figure 2G). To ensure that the number of macrophages injected is within the physiological range, the mice were administered 0.5×10^6^ cells, which represent half the number of resident peritoneal cavity macrophages. Consistent with our hypothesis, DNase-HCKO mice that received WT macrophages, but not DNase-deficient macrophages, had increased DNase activity, which was comparable with the WT mice (Figure 2H). Most importantly, these mice efficiently degraded the injected NETs (Figure 2I) and were associated with improved macrophage efferocytosis (Figure 2J). Overall, these data suggest that macrophages are critical for the NET-induced DNase response and play a dominant role in the clearance of locally generated NETs within the inflamed tissues.

### NETs Impair Macrophage Efferocytosis via Cleavage of the Efferocytic Receptor MerTK

**Figure 3.**
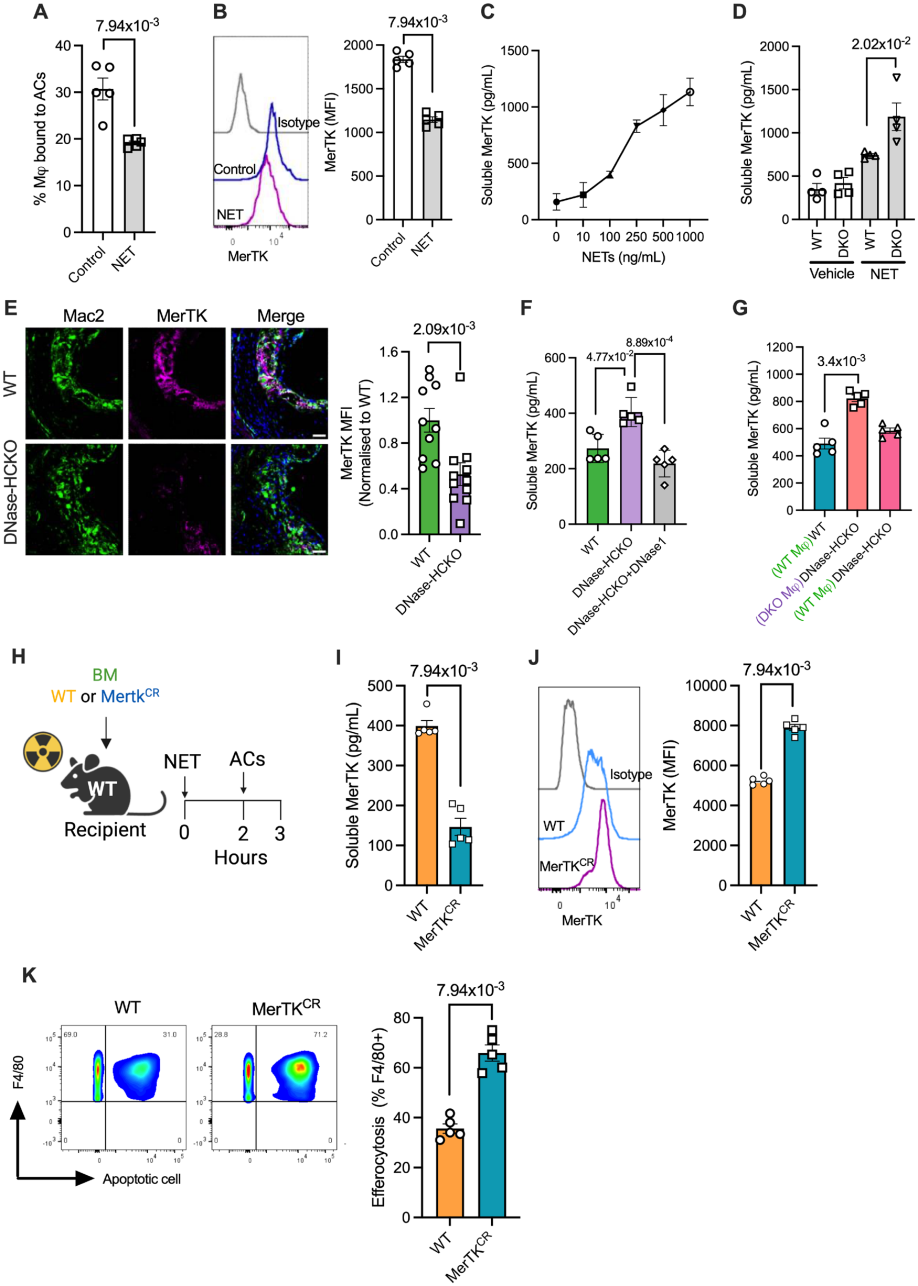
**Neutrophil extracellular traps (NETs) cleave MerTK to impair efferocytosis. A**, bone marrow–derived macrophages (BMDMs) were exposed to NETs (250 ng/mL), then incubated with cytochalasin D (1 µmol/L) for 30 minutes, followed by the addition of fluorescently labeled apoptotic cells (ACs). After washes, AC-binding efficiency was quantified by fluorescence microscopy. n=5 biological replicates. **B**, Quantification of cell surface MerTK levels in BMDMs treated with or without NETs for 2 hours. n=5 biological replicates. **C**, ELISA-based measurement of soluble MerTK in the cell culture supernatants of BMDMs exposed for 2 hours with indicated concentrations of NETs. n=3 biological replicates. **D**, Quantification of soluble MerTK in the cell culture supernatant of wild-type (WT) and double knockout (DKO) BMDMs exposed to vehicle or NETs (250 ng/mL) for 2 hours. n=4 biological replicates. **E**, Quantification of MerTK levels in lesional macrophages (Mac2+) by immunostaining of aortic root sections of 16-week Western diet–fed bone marrow chimeric WT and hematopoietic cell–specific DNase1/DNase1L3 knockout (DNase-HCKO) *Ldlr*^*−/−*^ mice. n=10 mice per group. **F**, ELISA-based analysis of soluble MerTK levels in the peritoneal lavage of bone marrow chimeric WT and DNase-HCKO mice 2 hours after intraperitoneal injection of NETs without or with DNase1. n=5 mice per group. **G**, As mentioned above, except that WT and DNase-HCKO mice were adoptively transferred either WT or DKO macrophages intraperitoneally 16 hours before injection of NETs. n=5 mice per group. **H**, Eight-week-old male *C57BL/6J* mice were lethally irradiated and injected with bone marrow cells from either WT or MerTK cleavage–resistant (MerTK^CR^) male mice. Six weeks later, bone marrow chimeric mice were injected with NETs (1 µg IP) and fluorescently labeled ACs 2 hours later. Peritoneal lavage was performed 1 hour later for analysis of (**I**) soluble MerTK by ELISA; (**J**) macrophage MerTK expression by flow cytometry, and (**K**) macrophage efferocytosis efficiency by flow cytometry. n=5 mice per group. The data are represented as mean±SEM. Data were tested for normal distribution using the Shapiro-Wilk test. *P* values were calculated using the Mann-Whitney *U* test (**A**, **B**, **E**, **I** through **K**) and the Kruskal-Wallis test with Dunn multiple comparisons correction (**D**, **F**, and **G**). Mφ indicates macrophage; and MFI, median fluorescence intensity.

Because efferocytosis is a multistep process involving an initial step of recognition and binding of ACs followed by a second step of internalization,^27^ we tested which of these broad processes are affected in the presence of NETs. To specifically test the efficiency of AC binding, macrophages exposed to either vehicle or NETs were incubated with cytochalasin D, an actin polymerization inhibitor that blocks engulfment,^28^ before addition of ACs. Interestingly, macrophages exposed to NETs were less efficient at binding ACs (Figure 3A). Because efferocytosis receptors are critical for recognition and binding of ACs, we profiled the cell surface expression levels of major efferocytosis receptors, including MerTK, Tim4 (T‑cell immunoglobulin and mucin domain containing 4), Axl (AXL receptor tyrosine kinase), and LRP-1 (low-density lipoprotein receptor–related protein 1), by flow cytometry. While the levels of Tim4, Axl, and LRP-1 were unaffected (Figure S2C), macrophages exposed to NETs demonstrated decreased cell surface expression of MerTK (Figure 3B), a dominant AC recognition receptor in macrophages in several tissues.^29^ Cell surface expression of MerTK is regulated by posttranslational modifications, including proteolytic cleavage of its ectodomain, resulting in the release of soluble MerTK, which is associated with loss of receptor function.^30^ Indeed, the levels of soluble MerTK were higher in the cell culture supernatants of macrophages incubated with NETs and showed a clear dose response (Figure 3C). Consistent with these findings, the accumulation of NETs when incubated with DNase-deficient macrophages (Figure 1B) was associated with increased generation of soluble MerTK (Figure 3D). Further supporting these in vitro data, the increased accumulation of NETs in the atherosclerotic plaques of DNase-HCKO mice (Figure 1I) was associated with decreased expression of MerTK in Mac2+ lesional macrophages (Figure 3E). Similarly, the increased persistence of NETs in the peritoneal cavity of DNase-HCKO mice (Figure 2E) was associated with increased soluble MerTK levels (Figure 3F), and this effect could be reversed by promoting NET degradation via administration of either DNase1 (Figure 3F) or the adoptive transfer of WT macrophages (Figure 3G). More importantly, the blockage of soluble MerTK production and the preservation of cell surface MerTK levels in all these experimental conditions prevented the NET-induced impairment of macrophage efferocytosis (Figure 2F and 2G).

We previously reported the identification of the cleavage site residues of MerTK,^30^ which were mutated to generate a MerTK^CR^ knockin mouse that was resistant to ADAM17 (a disintegrin and metalloproteinase 17)–mediated proteolysis and shedding.^31^ We used these MerTK^CR^ mice to address the question of whether NET-induced MerTK cleavage is a causal mechanism driving defective macrophage efferocytosis. In this context, we generated bone marrow chimeric C57BL/6 mice that were reconstituted with cells from either WT or MerTK^CR^ mice. Both groups of mice were injected with NETs followed by fluorescently labeled ACs (Figure 3H). As expected, the soluble MerTK levels in the peritoneal lavage of the MerTK^CR^ mouse were significantly lower compared with the WT group (Figure 3I). Consistent with the decreased generation of soluble MerTK, the MerTK^CR^ group showed increased cell surface MerTK levels in the F4/80^+^ peritoneal cavity macrophages (Figure 3J). Most importantly, the efferocytosis efficiency of MerTK^CR^ mice was significantly higher than the WT group (Figure 3K). Taken together, these data demonstrate that cleavage of MerTK and the loss of this key AC recognition receptor drives the NET-induced impairment of macrophage efferocytosis.

### NET-Associated HMGB1 Signals Mertk Cleavage

**Figure 4.**
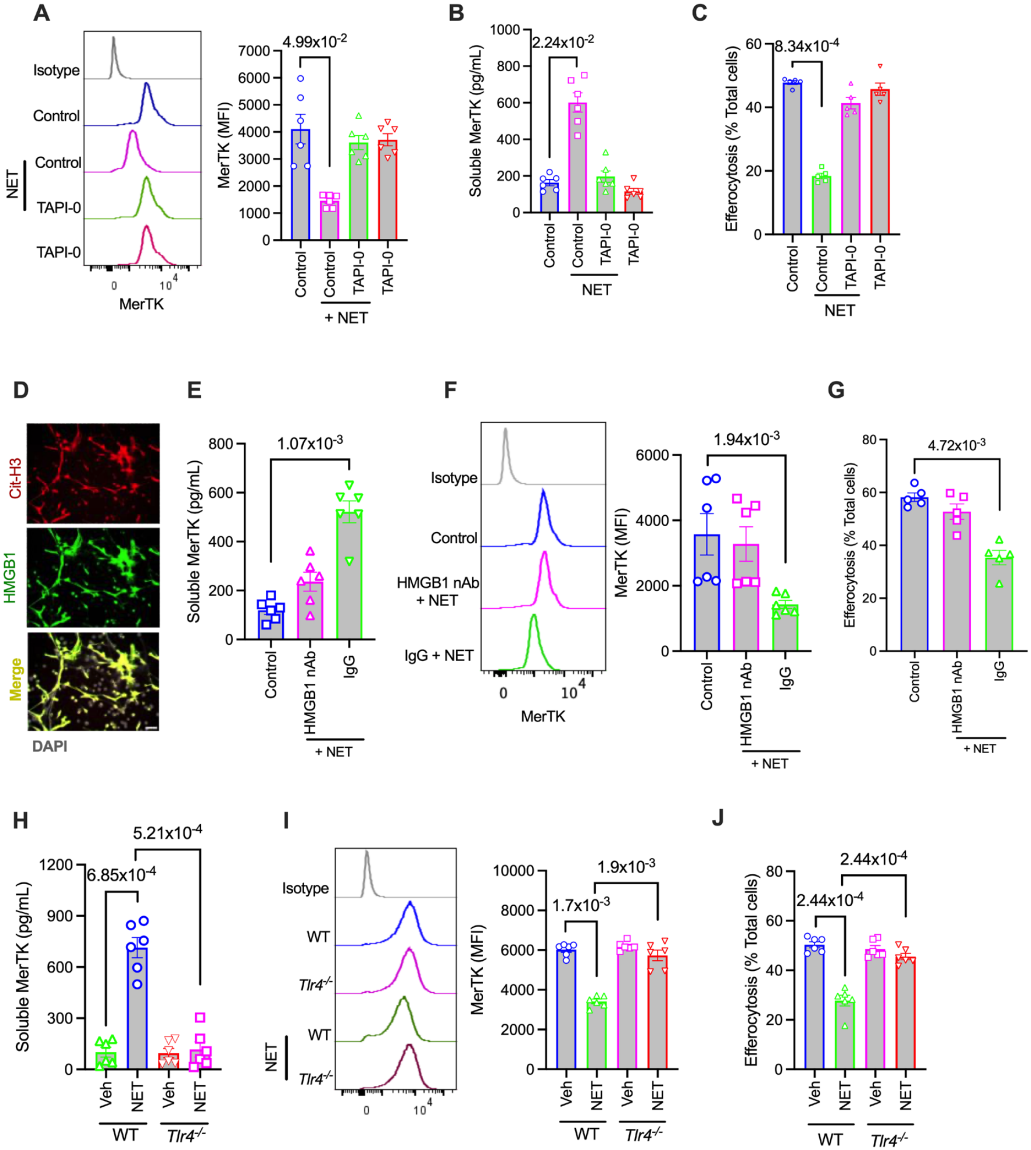
**Neutrophil extracellular trap (NET)–associated HMGB1 (high-mobility group box 1) triggers MerTK cleavage by TLR4-mediated activation of ADAM17 (a disintegrin and metalloproteinase 17). A**, Bone marrow–derived macrophages (BMDMs) were pretreated with TAPI-0 (10 µmol/L) or vehicle for 1 hour and then exposed to NETs for 2 hours. The cell surface MerTK levels were quantified by flow cytometry. n=6 biological replicates. **B**, As mentioned above, except that the culture supernatant was assayed by ELISA for soluble MerTK. n=6 biological replicates. **C**, Similar to **A**, except that TAPI-0 and NET-exposed BMDMs were incubated with fluorescently labeled apoptotic cells, and the quantification of efferocytosis efficiency was performed by microscopy. n=5 biological replicates. **D**, In vitro generated NETs were immunostained with anti–citrullinated histone H3 (CitH3) and anti-HMGB1 antibody and imaged by fluorescence microscopy. Nuclei were stained with DAPI (4′,6-diamidino-2-phenylindole). Scale bar, 50 µm. **E**, BMDMs were exposed to NETs in the presence of a HMGB1 neutralizing Ab (nAb) or control IgG. Soluble MerTK was measured in the culture supernatants. n=6 biological replicates. **F**, Similar to **E**, except that cell surface MerTK expression was quantified by flow cytometry. n=6 biological replicates. **G**, Efferocytosis efficiency was measured in BMDMs exposed to NETs in the presence of control IgG or HMGB1 nAb. n=6 biological replicates. **H**, Wild-type (WT) or *Tlr4*^*−/−*^ BMDMs were exposed to NETs, followed by quantification of soluble MerTK levels in the supernatant, and (**I**) MerTK expression on the cell surface. n=6 biological replicates. **J**, Quantification of efferocytosis in WT and *Tlr4*^*−/−*^ BMDMs exposed to vehicle or NETs. n=6 biological replicates. The data are represented as mean±SEM. *P* values were calculated using the Kruskal-Wallis test with Dunn multiple comparisons correction (**A** through **C** and **E** through **G**) or Aligned Rank Transform ANOVA (**H** through **J**). Ab indicates antibody; MFI, median fluorescence intensity; TAPI, TNF-α protease inhibitor; TLR, toll-like receptor; and Veh, vehicle.

We next explored the molecular mechanisms by which NETs trigger MerTK cleavage. First, we tested whether NETs activate ADAM17 to induce MerTK cleavage. Indeed, when macrophages were exposed to NETs in the presence of TAPI-0 (TNF-α processing inhibitor-0), an ADAM17 inhibitor,^30^ both the generation of soluble MerTK and the decrease in cell surface MerTK were abrogated (Figure 4A and 4B), along with preservation of macrophage efferocytosis efficiency (Figure 4C). These data demonstrate that NETs promote cleavage and shedding of MerTK via activation of the classical ADAM17 pathway.

We then examined how NETs activate macrophage ADAM17. Notably, NETs are known to activate TLR4 (toll-like receptor 4),^9,32^ which is upstream of the TRIF (TIR-domain-containing adaptor-inducing interferon-β)-ROS (reactive oxygen species)-p38 mitogen-activated protein kinase pathway that leads to ADAM17 activation.^30^ In this context, we set out to identify the specific NET-associated protein that activates TLR4. Interestingly, mass spectrometry analysis revealed HMGB1, a prominent TLR4 activator,^33^ as one of the dominant NET-associated proteins (Figure S3A). Both immunostaining (Figure 4D) and Western blotting (Figure S3B) confirmed the presence of HMGB1 on NETs. Furthermore, incubation of macrophages with recombinant HMGB1 led to MerTK cleavage and decreased efferocytosis efficiency (Figure S3C and S3D). We therefore tested whether NET-associated HMGB1 is relevant for MerTK cleavage. Interestingly, the incubation of macrophages with NETs in the presence of a HMGB1 neutralizing antibody blocked the production of NET-induced soluble MerTK (Figure 4E), enhanced cell surface MerTK (Figure 4F), and restored macrophage efferocytosis efficiency (Figure 4G). Consistent with these data, HMGB1-deficient NETs, derived from HL-60 cells subjected to siRNA-mediated knockdown of HMGB1 (Figure S3E), were unable to induce MerTK cleavage (Figure S3F) or impair efferocytosis in macrophages (Figure S3G). These data established HMGB1 as a NET-associated protein triggering MerTK cleavage. Finally, we tested whether HMGB1 signals via TLR4, a known activator of ADAM17, to promote MerTK cleavage. Indeed, macrophages from *Tlr4*^*−/−*^ mice were resistant to HMGB1-induced generation of Soluble MerTK, loss of cell surface MerTK, and impairment of efferocytosis (Figure S3H through S3J). More importantly, the lack of TLR4 in macrophages abrogated the ability of NETs to induce MerTK cleavage (Figure 4H and 4I) and block efferocytosis (Figure 4J). These data demonstrate that NET-associated HMGB1 is critical for TLR4-mediated activation of MerTK cleavage and impairment of macrophage efferocytosis.

### ATF4 Activation in ER-Stressed Atherogenic Macrophages Impairs Their NET-Induced DNase Response

**Figure 5.**
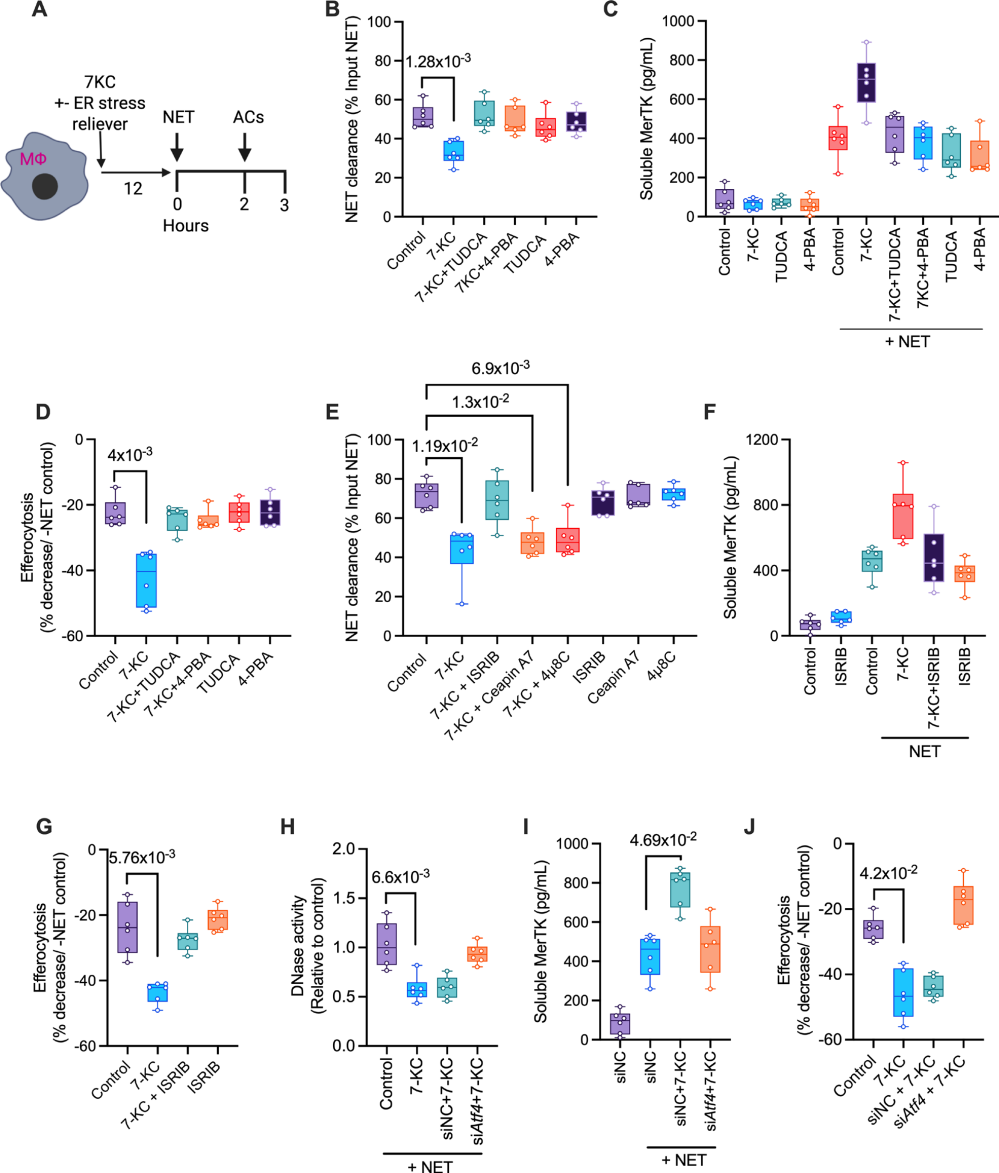
**ATF (activating transcription factor) 4 signaling in atherogenic macrophages impairs neutrophil extracellular trap (NET)–induced DNase response. A**, Bone marrow–derived macrophages (BMDMs) were incubated with 7-ketocholesterol (7-KC, 15 µmol/L) in the absence or presence of tauroursodeoxycholic acid (TUDCA; 3 mmol/L) or 4-phenylbutyric acid (4-PBA; 0.5 μM) for 18 hours. NETs (250 ng/mL), followed by fluorescently labeled apoptotic cells (ACs), were added to the culture. The supernatant was used for analysis of (**B**) DNase activity as a function of NET clearance by picogreen assay, and (**C**) soluble MerTK levels by ELISA, whereas the cells were used for (**D**) quantification of efferocytosis efficiency by fluorescence microscopy. n=6 biological replicates. **E**, BMDMs were incubated with 7-KC (15 µmol/L) along with either vehicle, integrated stress response inhibitor (ISRIB; 3 mmol/L), Ceapin A7 (1µM), or 4µ8C (100 µmol/L), for 18 hours, followed by exposure to NETs for 2 hours. The NET clearance efficiency was measured by the Picogreen assay in the supernatant. n=6 biological replicates. **F**, Quantification of soluble MerTK in the supernatants of vehicle or NET-exposed BMDMs pretreated with 7-KC in the absence or presence of ISRIB. n=6 biological replicates. **G**, Quantification of efferocytosis efficiency in vehicle or NET-exposed BMDMs pretreated with 7-KC in the absence or presence of ISRIB. n=6 biological replicates. **H**, BMDMs transfected with negative control siRNA (siNC) or ATF4 siRNA (si*Atf4*) were incubated with vehicle or 7-KC as indicated, followed by exposure to NETs for the measurement of DNase activity, and (**I**) soluble MerTK levels in the supernatant. n=6 biological replicates. **J**, Similar to **H**, except that NET-exposed macrophages were incubated with ACs for the quantification of efferocytosis efficiency. n=6 biological replicates. The data are represented as mean±SEM. *P* values were calculated using the Kruskal-Wallis test with Dunn multiple comparisons correction (**B** through **J**). ER indicates endoplasmic reticulum. Mφ indicates macrophage; siRNA, silencing RNA; and 4μ8C, 7-hydroxy-4-methyl-2-oxo-2H-1-benzopyran-8-carboxaldehyde.

Both proinflammatory M1-polarized and proreparative M2-polarized macrophages contain comparable levels of intracellular DNase1 and DNase1L3 (Figure S4A), and release a similar amount of DNase when exposed to NETs (Figure S4B). In contrast, ER-stressed lipid-loaded atherogenic macrophages generated by exposure to 7-ketocholesterol have impaired NET-induced DNase response.^18^ Extending these findings, we now show that the increased accumulation of NETs (Figure 5A and 5B) in this pathophysiologically relevant state mimicking macrophages in an atherogenic environment is associated with a trend towards increased MerTK cleavage (Figure 5C) and a statistically significant impairment in efferocytosis (Figure 5D), both of which could be reversed by ER stress relieving agents such as TUDCA and PBA (Figure 5C and 5D). To understand the specific ER stress signaling cascade that mediates the impairment in the NET-induced DNase response, 7-ketocholesterol–exposed macrophages were treated with either ISRIB, 4µ8C (7-hydroxy-4-methyl-2-oxo-2H-1-benzopyran-8-carboxaldehyde), or CeapinA7, specific inhibitors of the PERK, IRE1α (inositol-requiring enzyme 1 alpha), and ATF6 branch of the ER stress pathway, respectively, and the DNase activity in the supernatant was measured. Interestingly, the 7-ketocholesterol–induced impairment in NET clearance was reversed specifically in macrophages exposed to the PERK inhibitor ISRIB, but not when exposed to IRE1α or ATF6 inhibitor (Figure 5E). Consistent with these findings, ISRIB downregulated ATF4 levels in 7-ketocholesterol–exposed macrophages (Figure S4C), enhanced the NET-induced DNase response, and tended to lower MerTK cleavage (Figure 5F) and impairment of efferocytosis (Figure 5G). More importantly, similar findings were obtained in ATF4 knockdown macrophages (Figure 5H through 5J; Figure S4D), confirming that the PERK-ATF4 branch of the ER stress signaling cascade is critical for the NET-induced impairment of DNase response in foamy atherogenic macrophages.

### ISRIB Boosts the NET-Induced DNase Response and Promotes NET Clearance in Human and Mouse Atherosclerotic Explant Tissue

**Figure 6.**
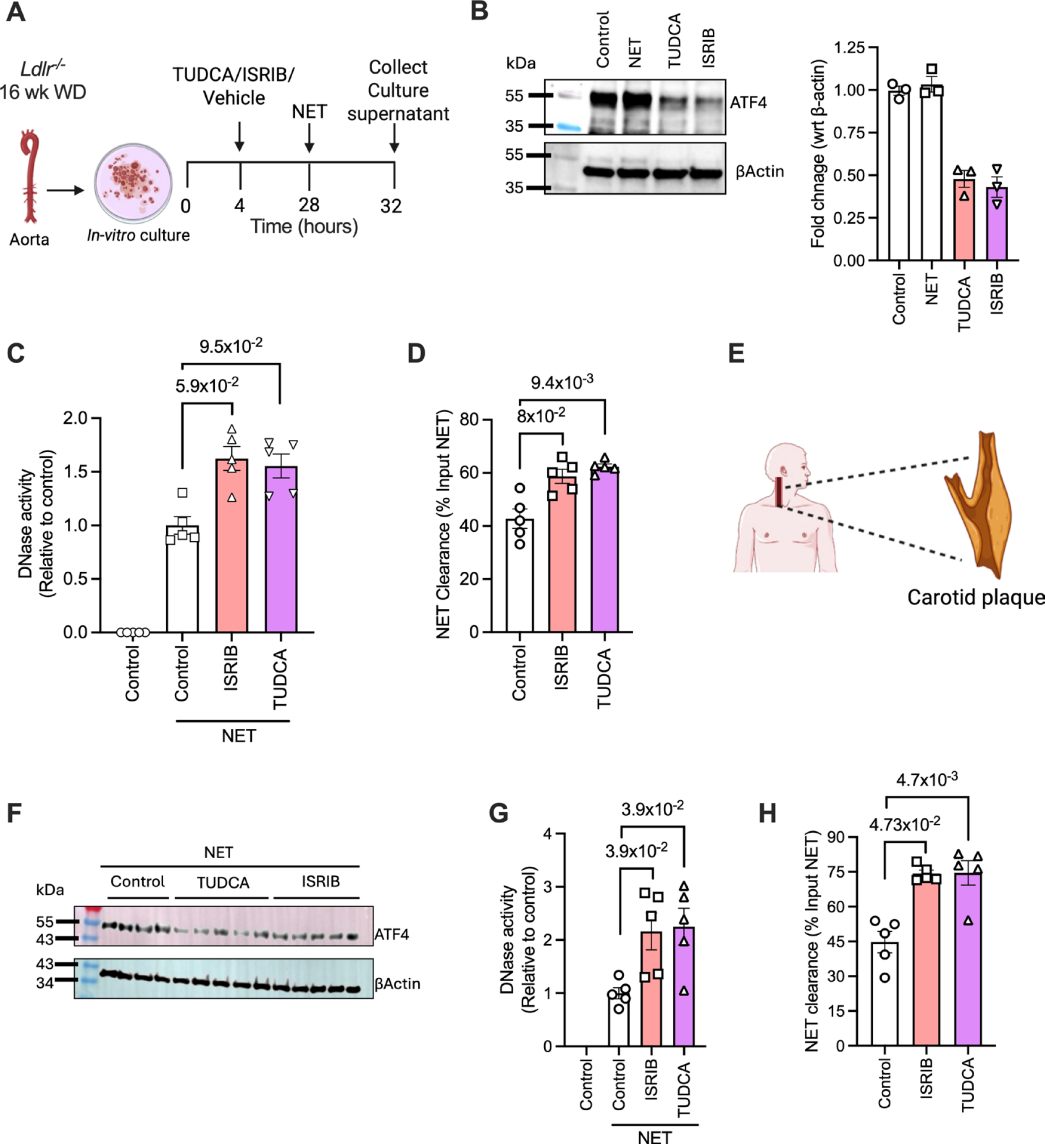
**Integrated stress response inhibitor (ISRIB) increases neutrophil extracellular trap (NET)–induced DNase response in mouse and human atherosclerotic plaques. A**, Aorta harvested from 16-week Western diet (WD)–fed *Ldlr*^*−/−*^ mice were cultured as explants and treated with either vehicle, tauroursodeoxycholic acid (TUDCA), or ISRIB (3 mmol/L) for 18 hours, followed by exposure to NETs for 4 hours. Tissue lysates were used for (**B**) immunoblotting of ATF (activating transcription factor) 4, whereas the culture supernatant was tested for (**C**) DNase activity and (**D**) NET clearance efficiency. n=4 mice per group. **E** through **H**, Similar to (**A** through **D**), except that human carotid endarterectomy tissues were treated with vehicle, TUDCA, or ISRIB, followed by exposure to NETs for analysis of (**F**) tissue ATF4 levels by immunoblotting; (**G**) DNase activity, and (**H**) NET clearance efficiency in the culture supernatant. n=5. The data are represented as mean±SEM. *P* values were calculated using the Kruskal-Wallis test with Dunn multiple comparisons correction (**C**, **D**, **G**, and **H**). WT indicates wild-type.

We next questioned whether these findings are relevant in the pathological context of mouse and human atherosclerosis. Towards this end, the atherosclerotic aorta was harvested from *Ldlr*^*−/−*^ mice that were fed a Western diet for 16 weeks and cut into smaller fragments, followed by culturing ex vivo as explants. The tissue was treated with either vehicle, ISRIB, or TUDCA for 4 hours before the addition of NETs, and the supernatant was collected for measurement of DNase activity and NET clearance (Figure 6A). First, immunoblotting of tissue extracts confirmed that treatment with both ISRIB and TUDCA decreased the ATF4 levels (Figure 6B). Most importantly, both ISRIB and TUDCA led to improvement in the NET-induced DNase response (Figure 6C), which was associated with accelerated clearance of NETs (Figure 6D). Similar to our findings above, the inhibition of ATF4 in atherosclerotic tissue explant culture from patients undergoing carotid endarterectomy by treatment with ISRIB or TUDCA (Figure 6E-F) led to an increase in NET-induced DNase activity in the supernatant (Figure 6G) and enhanced clearance of NETs (Figure 6H). Together, these findings demonstrate that activation of the ATF4 branch of the ER stress signaling pathway mediates the impairment of the protective NET-induced DNase response in atherosclerosis.

### ISRIB Improves the Vascular DNase Response In Vivo and Stabilizes Advanced Atherosclerotic Plaques

**Figure 7.**
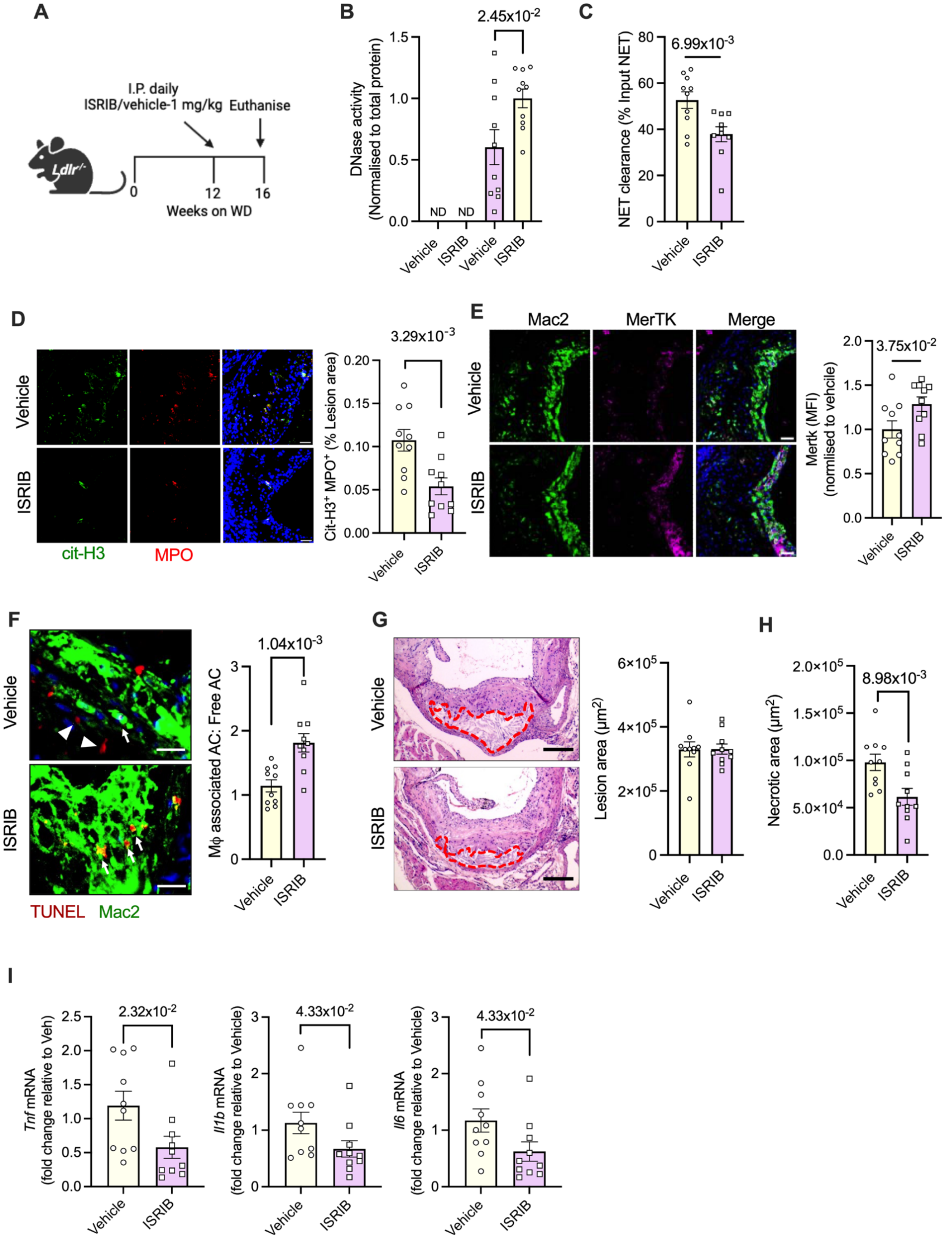
**Integrated stress response inhibitor (ISRIB) enhances vascular DNase activity and neutrophil extracellular trap (NET) clearance in murine atherosclerosis. A**, Ten-week-old female *Ldlr*^*−/−*^ mice were fed a Western diet (WD) for 16 weeks. During the final 4 weeks, one group of mice received ISRIB (1 mg/kg IP) daily, whereas the other group received vehicle. **B**, Aortic explants from vehicle and ISRIB-treated mice were exposed to NETs for measurement of DNase activity, and (**C**) NET clearance efficiency, in the supernatants. **D**, Aortic root sections were immunostained with anti-MPO (myeloperoxidase) and anticitrullinated histone H3 (CitH3) antibody for quantification of lesional NET levels, or (**E**) MerTK levels in lesional Mac2+ macrophages. **F**, Aortic root sections were labeled with terminal deoxynucleotidyl transferase dUTP nick-end labeling (TUNEL; red) and immunostained with anti-Mac2 antibody (green) for measurement of lesional in situ efferocytosis efficiency as a ratio of macrophage-associated apoptotic cells (ACs):free-lying ACs. White arrows indicate TUNEL+ ACs associated with a macrophage. White arrowheads show free-lying TUNEL+ cells. **G**, Hematoxylin and eosin–stained aortic root sections were used for quantification of total lesion area, and (**H**) necrotic area, in vehicle and ISRIB-treated mice. The necrotic regions are demarcated by the red dashed lines. **I**, Quantitative polymerase chain reaction–based analysis of *Tnf*, *Il1b*, and *Il6*, in vascular tissues obtained from vehicle and ISRIB-treated mice. n=10 mice per group. The data are represented as mean±SEM. Data were tested for normal distribution using Shapiro-Wilk test. *P* values were calculated using unpaired *t* test (**B** through **F**, and **H**), Mann-Whitney *U* test (**G** and **I**). AC indicates apoptotic cell; MFI, median fluorescence intensity; Mφ, macrophage; and ND, not detected.

Based on the ex vivo data above, we questioned whether inhibition of ATF4 with ISRIB could enhance DNase secretion by ER-stressed lesional macrophages in vivo to promote the clearance of NETs in advanced atherosclerotic plaques. To address this question, *Ldlr*^*−/−*^ mice were fed a Western diet for 12 weeks to induce the generation of advanced atherosclerotic plaques and were then administered either ISRIB (1 mg/kg intraperitoneal) or vehicle for the next 4 weeks while continuing a Western diet (Figure 7A). Consistent with inhibition of PERK signaling, ISRIB-treated mice had lower ATF4 levels in the aorta (Figure S5A). Plasma DNase activity and NET levels (Figure S5B and S5C) and the basal aortic tissue DNase activity (Figure S5D) were not different between the 2 groups of mice, suggesting that ATF4 inhibition does not affect the total DNase levels in blood and tissues. However, aortic explants from the ISRIB-treated group demonstrated increased release of DNase in the supernatant in response to NETs (Figure 7B) and increased efficiency of clearance of NETs (Figure 7C), suggesting that ATF4 inhibition by ISRIB increases the NET-induced DNase response in atherosclerosis. Consistent with these ex vivo data, the atherosclerotic lesional NET content was significantly lower in the ISRIB-treated mice as compared with the vehicle-treated mice (Figure 7D). Because ISRIB does not affect cholesterol-induced neutrophil NETosis (Figure S5E), these data demonstrate that ATF4 inhibition in vivo increases the clearance of NETs via enhanced DNase-mediated degradation. More importantly, as predicted, the decrease in lesional NET content in the ISRIB-treated mice was associated with both higher MerTK levels in lesional macrophages (Figure 7E) and increased efferocytosis efficiency (Figure 7F). ISRIB treatment had no effect on the total atherosclerotic lesion area (Figure 7G), which is consistent with the lack of difference in plasma cholesterol, triglycerides, and glucose between the vehicle- and ISRIB-treated mice (Figure S5F through S5I). However, interestingly, the ISRIB-treated mice showed significant plaque remodeling as demonstrated by the decrease in plaque necrosis (Figure 7H) and increased intimal collagen content (Figure S5J), which is consistent with the improvement in lesional macrophage efferocytosis in these mice. Finally, the ISRIB-treated mice showed a decrease in the mRNA levels of key proinflammatory cytokines, including *Tnf*, *Il1b*, and *Il6* (Figure 7I). These data suggest that ATF4 inhibition mediated by ISRIB could promote plaque stabilization in advanced atherosclerosis via improved macrophage DNase release, NET degradation, and preservation of macrophage efferocytosis efficiency.

## Discussion

Our study highlights the critical role of macrophages in the clearance of NETs in inflamed tissues through the release of DNases. We demonstrate that ER stress–mediated activation of the PERK-ATF4 pathway impairs the NET-induced DNase response in lesional macrophages, resulting in defective NET clearance and their persistence in atherosclerosis. Notably, this defect can be reversed with the PERK inhibitor ISRIB. These data highlight impaired NET clearance as a key driver of NET persistence in atherosclerosis. Finally, we demonstrate that NETs promote the cleavage and shedding of MerTK on macrophages, leading to decreased lesional efferocytosis. Collectively, these findings provide a mechanistic understanding of NET persistence in atherosclerosis and its role in driving plaque necrosis and instability through cellular cross-talk.

NETosis is induced in the atherosclerotic plaque by multiple factors, including cholesterol crystals,^9^ oxidative stress,^34^ and inflammatory cytokines, such as CCL7^8^ and IL1β.^35^ In addition, mutations driving clonal hematopoiesis of indeterminate potential are associated with enhanced NETosis susceptibility.^11–13^ Inhibition of NETosis by targeting neutrophil elastase,^36^ PAD4 (peptidylarginine deiminase 4),^37^ MPO,^38^ or the blockage of NET-associated components such as histones,^32^ leads to decreased atherosclerosis progression. Similarly, degradation of NETs by administration of DNases is associated with decreased atherosclerosis and plaque stabilization.^18,39^ Therefore, NETosis inhibitors as well as novel synthetic DNases with high in vivo stability are highly sought after as therapeutic agents. However, NETs are important for host defense against pathogens and thus long-term inhibition of NETosis, such as that will be required for preventing atherosclerotic complications, is likely to be associated with compromised host defense and increased risk of infections, as already noted, with other anti-inflammatory therapies, such as anti-IL1β and colchicine.^40,41^ In this context, our discovery of fundamental molecular mechanisms that disrupt the physiological NET-induced DNase response, such as ER stress in atherosclerotic plaque macrophages, opens new therapeutic avenues via targeting these pathways.

Lesional cells, including plaque macrophages, experience ER stress due to a combination of lipid accumulation, oxidative stress, and exposure to inflammatory mediators within the atherosclerotic plaque niche. ER stress perturbs macrophage function, and when excessive, triggers cell death.^42^ Chemical chaperones such as 4-phenylbutyric acid and TUDCA relieve ER stress in atherosclerosis and are associated with decreased plaque progression and plaque stabilization.^43^ Because ER stress is a protective physiological response required for homeostatic functions, including insulin secretion by pancreatic β cells,^44^ antibody production by plasma cells,^45^ and dendritic cell differentiation,^46^ the use of chemical chaperones, such as PBA and TUDCA, leads to on-target adverse effects that hinder its clinical use. Therefore, our identification of the PERK-ATF4 branch of the ER stress signaling cascade as the specific trigger that impairs the NET-induced DNase response in lipid-loaded macrophages is a step change for the development of targeted therapeutic strategies. Indeed, we show that inhibition of PERK signaling using ISRIB enhances vascular DNase activity, decreases NET accumulation, and promotes plaque stabilization by decreasing lesional necrosis and enhancing collagen deposition. Given that ISRIB partially inhibits PERK signaling, it allows tempering or reshaping the aberrant ER stress responses to physiological levels and thus minimizes on-target toxicities associated with the abolishment of ER stress signaling.^47^ Importantly, our demonstration of conservation of these mechanistic pathways in human atherosclerosis suggests that ISRIB and similar new classes of drugs, complemented by cell- and tissue-targeted nanomedicines, provide a window of opportunity for therapeutic targeting of hyperactivated ER stress signaling cascades in chronic diseases such as atherosclerosis.^48,49^

There is extensive evidence that NETs increase plaque instability by decreasing fibrous cap thickness and increasing plaque necrosis.^18^ The NET-associated decrease in fibrous cap thickness is driven by a combination of NET-induced smooth muscle apoptosis^8^ and matrix metalloprotease-mediated degradation of extracellular matrix.^8^ In contrast, the mechanisms driving plaque necrosis are not well defined, although an increase in NET-induced lesional cell death has been implicated. In addition, NETs can increase proinflammatory macrophage polarization as well as block the generation of proresolving M2-like macrophages,^32,50^ which could accelerate atherosclerosis progression. However, whether NETs affect lesional efferocytosis, a major driver of atherosclerotic plaque necrosis, was unknown. Previous evidence from acute inflammatory disease settings, such as sepsis^51^ suggested that NETs could impair efferocytosis. Indeed, our findings highlight that NETs decrease macrophage efferocytosis efficiency by activating ADAM17-mediated cleavage and shedding of the dominant efferocytosis receptor MerTK. Although MerTK cleavage is a causal mechanism in driving plaque necrosis in advanced atherosclerosis,^19^ the pathophysiologically relevant inducers of MerTK cleavage in the context of atherosclerosis were not known. Recently, IL1β was described as an inducer of MerTK cleavage.^14^ Adding to this repertoire, our findings establish NETs and NET-associated HMGB1 as an atherosclerotic plaque-relevant inducer of MerTK cleavage. These findings raise the interesting possibility of targeting HMGB1 to preserve lesional efferocytosis efficiency. Moreover, because MerTK cleavage is a central mechanism in regulating plaque necrosis, therapeutic strategies that stabilize MerTK could be hugely beneficial in promoting plaque stabilization.

Although our findings provide mechanistic insights into macrophage-mediated NET clearance and its disruption in atherosclerosis, some limitations should be noted. First, we used a genetic model to delete DNases in hematopoietic cells and impair NET clearance. However, these enzymes may have additional cell type–specific and cell-intrinsic roles that could contribute in part to the observed plaque phenotypes. Second, our study primarily relies on mouse models, and while we demonstrate conservation of key mechanisms in human tissues, further validation in human in vivo systems is needed. Third, the effect of ISRIB was assessed in the context of plaque stability, but its long-term safety and efficacy in the setting of chronic cardiovascular disease remain to be determined.

In summary, our study advances several new concepts including (1) the establishment of macrophages as the major producer of DNases and as a critical determinant of the efficiency of NET clearance in tissues; (2) the demonstration that activation of PERK signaling is a key pathogenic mechanism impairing the NET-induced DNase response in atherogenic macrophages; and (3) the demonstration that NETs directly impair macrophage efferocytosis efficiency by cleavage of MerTK. These findings highlight several novel therapeutic strategies to enhance the clearance of lesional NETs and promote atherosclerotic plaque stabilization. Because NETs exacerbate the complications of atherosclerotic cardiovascular diseases such as myocardial infarction and stroke^52,53^ as well as play a pathogenic role in several chronic inflammatory diseases, our findings could be broadly relevant for the prevention NET-induced inflammation and tissue damage in these settings.

## ARTICLE INFORMATION

### Acknowledgments

The authors sincerely acknowledge Dr Santosh R. Sukka and Professor Ira Tabas, Columbia University Medical Center, NY, for providing bones from MerTK cleavage–resistant mice. *Tlr4*^*−/−*^ mice were kindly provided by Prof Shizuo Akira, Osaka University, through a Material Transfer Agreement with The Francis Crick Institute, United Kingdom.

### Sources of Funding

This study was supported by funding from the British Heart Foundation (PG/22/11226), UKRI-BBSRC (UK Research and Innovation - Biotechnology and Biological Sciences Research Council, BB/Y513143/1), and Barts Charity (MGU0459), awarded to M. Subramanian. T. Vartak was supported by a University College Dublin Ad Astra PhD studentship. C. Godson and E. Brennan are supported by Science Foundation Ireland Awards (21/US/3751) and by the UCD Foundation. E. Brennan and M. O’Donohoe are supported by a University College Dublin School of Medicine COINTREAU award.

### Disclosures

None.

## Supplementary Material



## References

[1] Martin SS Aday AW Almarzooq ZI Anderson CAM Arora P Avery CL Baker-Smith CM Barone Gibbs B Beaton AZ Boehme AK; American Heart Association Council on Epidemiology and Prevention Statistics Committee and Stroke Statistics Subcommittee. 2024 heart disease and stroke statistics: a report of US and global data from the American Heart Association. Circulation. 2024;149:e347–e913. doi: 10.1161/CIR.0000000000001209 38264914 10.1161/CIR.0000000000001209 PMC12146881

[2] Vaduganathan M Mensah GA Turco JV Fuster V Roth GA. The global burden of cardiovascular diseases and risk: a compass for future health. J Am Coll Cardiol. 2022;80:2361–2371. doi: 10.1016/j.jacc.2022.11.005 36368511 10.1016/j.jacc.2022.11.005

[3] Ridker PM. Residual inflammatory risk: addressing the obverse side of the atherosclerosis prevention coin. Eur Heart J. 2016;37:1720–1722. doi: 10.1093/eurheartj/ehw024 26908943 10.1093/eurheartj/ehw024

[4] Doran AC Yurdagul A Jr Tabas I. Efferocytosis in health and disease. Nat Rev Immunol. 2020;20:254–267. doi: 10.1038/s41577-019-0240-6 31822793 10.1038/s41577-019-0240-6 PMC7667664

[5] Kasikara C Doran AC Cai B Tabas I. The role of non-resolving inflammation in atherosclerosis. J Clin Invest. 2018;128:2713–2723. doi: 10.1172/JCI97950 30108191 10.1172/JCI97950 PMC6025992

[6] Doring Y Libby P Soehnlein O. Neutrophil extracellular traps participate in cardiovascular diseases: recent experimental and clinical insights. Circ Res. 2020;126:1228–1241. doi: 10.1161/CIRCRESAHA.120.315931 32324499 10.1161/CIRCRESAHA.120.315931 PMC7185047

[7] Westerterp M Fotakis P Ouimet M Bochem AE Zhang H Molusky MM Wang W Abramowicz S la Bastide-van Gemert S Wang N. Cholesterol efflux pathways suppress inflammasome activation, NETosis, and atherogenesis. Circulation. 2018;138:898–912. doi: 10.1161/CIRCULATIONAHA.117.032636 29588315 10.1161/CIRCULATIONAHA.117.032636 PMC6160368

[8] Silvestre-Roig C Braster Q Wichapong K Lee EY Teulon JM Berrebeh N Winter J Adrover JM Santos GS Froese A. Externalized histone H4 orchestrates chronic inflammation by inducing lytic cell death. Nature. 2019;569:236–240. doi: 10.1038/s41586-019-1167-6 31043745 10.1038/s41586-019-1167-6 PMC6716525

[9] Warnatsch A Ioannou M Wang Q Papayannopoulos VI. Neutrophil extracellular traps license macrophages for cytokine production in atherosclerosis. Science. 2015;349:316–320. doi: 10.1126/science.aaa8064 26185250 10.1126/science.aaa8064 PMC4854322

[10] Zhu Y Wang T Yang Y Wang Z Chen X Wang L Niu R Sun Z Zhang C Luo Y. Low shear stress exacerbates atherosclerosis by inducing the generation of neutrophil extracellular traps via Piezo1-mediated mechanosensation. Atherosclerosis. 2024;391:117473. doi: 10.1016/j.atherosclerosis.2024.117473 38412763 10.1016/j.atherosclerosis.2024.117473

[11] Dou H Kotini A Liu W Fidler T Endo-Umeda K Sun X Olszewska M Xiao T Abramowicz S Yalcinkaya M. Oxidized phospholipids promote NETosis and arterial thrombosis in LNK(SH2B3) deficiency. Circulation. 2021;144:1940–1954. doi: 10.1161/CIRCULATIONAHA.121.056414 34846914 10.1161/CIRCULATIONAHA.121.056414 PMC8663540

[12] Huerga Encabo H Aramburu IV Garcia-Albornoz M Piganeau M Wood H Song A Ferrelli A Sharma A Minutti CM Domart MC. Loss of TET2 in human hematopoietic stem cells alters the development and function of neutrophils. Cell Stem Cell. 2023;30:781.e9–799.e9. doi: 10.1016/j.stem.2023.05.004 37267914 10.1016/j.stem.2023.05.004 PMC12356769

[13] Wolach O Sellar RS Martinod K Cherpokova D McConkey M Chappell RJ Silver AJ Adams D Castellano CA Schneider RK. Increased neutrophil extracellular trap formation promotes thrombosis in myeloproliferative neoplasms. Sci Transl Med. 2018;10:eaan8292. doi: 10.1126/scitranslmed.aan8292 29643232 10.1126/scitranslmed.aan8292 PMC6442466

[14] Liu W Hardaway BD Kim E Pauli J Wettich JL Yalcinkaya M Hsu CC Xiao T Reilly MP Tabas I. Inflammatory crosstalk impairs phagocytic receptors and aggravates atherosclerosis in clonal hematopoiesis in mice. J Clin Invest. 2024;135:e182939. doi: 10.1172/JCI182939 39531316 10.1172/JCI182939 PMC11684819

[15] Papayannopoulos V. Neutrophil extracellular traps in immunity and disease. Nat Rev Immunol. 2018;18:134–147. doi: 10.1038/nri.2017.105 28990587 10.1038/nri.2017.105

[16] Englert H Göbel J Khong D Omidi M Wolska N Konrath S Frye M Mailer RK Beerens M Gerwers JC. Targeting NETs using dual-active DNase1 variants. Front Immunol. 2023;14:1181761. doi: 10.3389/fimmu.2023.1181761 37287977 10.3389/fimmu.2023.1181761 PMC10242134

[17] Jimenez-Alcazar M Rangaswamy C Panda R Bitterling J Simsek YJ Long AT Bilyy R Krenn V Renne C Renne T. Host DNases prevent vascular occlusion by neutrophil extracellular traps. Science. 2017;358:1202–1206. doi: 10.1126/science.aam8897 29191910 10.1126/science.aam8897

[18] Dhawan UK Bhattacharya P Narayanan S Manickam V Aggarwal A Subramanian M. Hypercholesterolemia impairs clearance of neutrophil extracellular traps and promotes inflammation and atherosclerotic plaque progression. Arterioscler Thromb Vasc Biol. 2021;41:2598–2615. doi: 10.1161/ATVBAHA.120.316389 34348488 10.1161/ATVBAHA.120.316389 PMC8454501

[19] Cai B Thorp EB Doran AC Sansbury BE Daemen MJ Dorweiler B Spite M Fredman G Tabas I. MerTK receptor cleavage promotes plaque necrosis and defective resolution in atherosclerosis. J Clin Invest. 2017;127:564–568. doi: 10.1172/JCI90520 28067670 10.1172/JCI90520 PMC5272169

[20] Hoshino K Takeuchi O Kawai T Sanjo H Ogawa T Takeda Y Takeda K Akira S. Cutting edge: Toll-like receptor 4 (TLR4)-deficient mice are hyporesponsive to lipopolysaccharide: evidence for TLR4 as the LPS gene product. J Immunol. 1999;162:3749–3752. doi: 10.1093/jimmunol/197.7.2563. 10201887

[21] Bhattacharya P Dhawan UK Hussain MT Singh P Bhagat KK Singhal A Austin-Williams S Sengupta S Subramanian M. Efferocytes release extracellular vesicles to resolve inflammation and tissue injury via prosaposin-GPR37 signaling. Cell Rep. 2023;42:112808. doi: 10.1016/j.celrep.2023.112808 37436891 10.1016/j.celrep.2023.112808

[22] Brennan E Wang B McClelland A Mohan M Marai M Beuscart O Derouiche S Gray S Pickering R Tikellis C. Protective effect of let-7 miRNA family in regulating inflammation in diabetes-associated atherosclerosis. Diabetes. 2017;66:2266–2277. doi: 10.2337/db16-1405 28487436 10.2337/db16-1405

[23] de Gaetano M Crean D Barry M Belton O. M1- and M2-type macrophage responses are predictive of adverse outcomes in human atherosclerosis. Front Immunol. 2016;7:275. doi: 10.3389/fimmu.2016.00275 27486460 10.3389/fimmu.2016.00275 PMC4949256

[24] Kojima Y Weissman IL Leeper NJ. The role of efferocytosis in atherosclerosis. Circulation. 2017;135:476–489. doi: 10.1161/CIRCULATIONAHA.116.025684 28137963 10.1161/CIRCULATIONAHA.116.025684 PMC5302553

[25] Yurdagul A Jr. Assessing efferocytosis in atherosclerotic lesions in situ. Methods Mol Biol. 2022;2419:561–567. doi: 10.1007/978-1-0716-1924-7_34 35237988 10.1007/978-1-0716-1924-7_34

[26] Subramanian M Thorp E Tabas I. Identification of a non-growth factor role for GM-CSF in advanced atherosclerosis: promotion of macrophage apoptosis and plaque necrosis through IL-23 signaling. Circ Res. 2015;116:e13–e24. doi: 10.1161/CIRCRESAHA.116.304794 25348165 10.1161/CIRCRESAHA.116.304794 PMC4297527

[27] Mehrotra P Ravichandran KS. Drugging the efferocytosis process: concepts and opportunities. Nat Rev Drug Discov. 2022;21:601–620. doi: 10.1038/s41573-022-00470-y 35650427 10.1038/s41573-022-00470-y PMC9157040

[28] Casella JF Flanagan MD Lin S. Cytochalasin D inhibits actin polymerization and induces depolymerization of actin filaments formed during platelet shape change. Nature. 1981;293:302–305. doi: 10.1038/293302a0 7196996 10.1038/293302a0

[29] Dhawan UK Singhal A Subramanian M. Dead cell and debris clearance in the atherosclerotic plaque: mechanisms and therapeutic opportunities to promote inflammation resolution. Pharmacol Res. 2021;170:105699. doi: 10.1016/j.phrs.2021.105699 34087352 10.1016/j.phrs.2021.105699

[30] Thorp E Vaisar T Subramanian M Mautner L Blobel C Tabas I. Shedding of the Mer tyrosine kinase receptor is mediated by ADAM17 protein through a pathway involving reactive oxygen species, protein kinase Cδ, and p38 mitogen-activated protein kinase (MAPK). J Biol Chem. 2011;286:33335–33344. doi: 10.1074/jbc.M111.263020 21828049 10.1074/jbc.M111.263020 PMC3190938

[31] Cai B Thorp EB Doran AC Subramanian M Sansbury BE Lin CS Spite M Fredman G Tabas I. MerTK cleavage limits proresolving mediator biosynthesis and exacerbates tissue inflammation. Proc Natl Acad Sci USA. 2016;113:6526–6531. doi: 10.1073/pnas.1524292113 27199481 10.1073/pnas.1524292113 PMC4988577

[32] Tsourouktsoglou TD Warnatsch A Ioannou M Hoving D Wang Q Papayannopoulos V. Histones, DNA, and citrullination promote neutrophil extracellular trap inflammation by regulating the localization and activation of TLR4. Cell Rep. 2020;31:107602. doi: 10.1016/j.celrep.2020.107602 32375035 10.1016/j.celrep.2020.107602

[33] Saenz R Futalan D Leutenez L Eekhout F Fecteau JF Sundelius S Sundqvist S Larsson M Hayashi T Minev B. TLR4-dependent activation of dendritic cells by an HMGB1-derived peptide adjuvant. J Transl Med. 2014;12:211. doi: 10.1186/1479-5876-12-211 25123824 10.1186/1479-5876-12-211 PMC4261565

[34] Wang Y Wang W Wang N Tall AR Tabas I. Mitochondrial oxidative stress promotes atherosclerosis and neutrophil extracellular traps in aged mice. Arterioscler Thromb Vasc Biol. 2017;37:e99–e107. doi: 10.1161/ATVBAHA.117.309580 28596373 10.1161/ATVBAHA.117.309580 PMC5535797

[35] Yalcinkaya M Fotakis P Liu W Endo-Umeda K Dou H Abramowicz S Xiao T Libby P Wang N Tall AR. Cholesterol accumulation in macrophages drives NETosis in atherosclerotic plaques via IL-1β secretion. Cardiovasc Res. 2023;119:969–981. doi: 10.1093/cvr/cvac189 36537208 10.1093/cvr/cvac189 PMC10153645

[36] Shi Y Dong M Wu Y Gong F Wang Z Xue L Su Z. An elastase-inhibiting, plaque-targeting and neutrophil-hitchhiking liposome against atherosclerosis. Acta Biomater. 2024;173:470–481. doi: 10.1016/j.actbio.2023.11.020 37984628 10.1016/j.actbio.2023.11.020

[37] Molinaro R Yu M Sausen G Bichsel CA Corbo C Folco EJ Lee GY Liu Y Tesmenitsky Y Shvartz E. Targeted delivery of protein arginine deiminase-4 inhibitors to limit arterial intimal NETosis and preserve endothelial integrity. Cardiovasc Res. 2021;117:2652–2663. doi: 10.1093/cvr/cvab074 33751034 10.1093/cvr/cvab074 PMC8783386

[38] Chen W Tumanov S Kong SMY Cheng D Michaelsson E Bongers A Power C Ayer A Stocker R. Therapeutic inhibition of MPO stabilizes pre-existing high risk atherosclerotic plaque. Redox Biol. 2022;58:102532. doi: 10.1016/j.redox.2022.102532 36375379 10.1016/j.redox.2022.102532 PMC9663534

[39] Josefs T Barrett TJ Brown EJ Quezada A Wu X Voisin M Amengual J Fisher EA. Neutrophil extracellular traps promote macrophage inflammation and impair atherosclerosis resolution in diabetic mice. JCI Insight. 2020;5:e134796. doi: 10.1172/jci.insight.134796 32191637 10.1172/jci.insight.134796 PMC7205252

[40] Imazio M Lazaros G Gattorno M LeWinter M Abbate A Brucato A Klein A. Anti-interleukin-1 agents for pericarditis: a primer for cardiologists. Eur Heart J. 2022;43:2946–2957. doi: 10.1093/eurheartj/ehab452 34528670 10.1093/eurheartj/ehab452 PMC9375710

[41] McKnight AH Katzenberger DR Britnell SR. Colchicine in acute coronary syndrome: a systematic review. Ann Pharmacother. 2021;55:187–197. doi: 10.1177/1060028020942144 32659104 10.1177/1060028020942144

[42] Devries-Seimon T Li Y Yao PM Stone E Wang Y Davis RJ Flavell R Tabas I. Cholesterol-induced macrophage apoptosis requires ER stress pathways and engagement of the type A scavenger receptor. J Cell Biol. 2005;171:61–73. doi: 10.1083/jcb.200502078 16203857 10.1083/jcb.200502078 PMC2171235

[43] Erbay E Babaev VR Mayers JR Makowski L Charles KN Snitow ME Fazio S Wiest MM Watkins SM Linton MF. Reducing endoplasmic reticulum stress through a macrophage lipid chaperone alleviates atherosclerosis. Nat Med. 2009;15:1383–1391. doi: 10.1038/nm.2067 19966778 10.1038/nm.2067 PMC2790330

[44] Rabhi N Salas E Froguel P Annicotte JS. Role of the unfolded protein response in beta cell compensation and failure during diabetes. J Diabetes Res. 2014;2014:795171. doi: 10.1155/2014/795171 24812634 10.1155/2014/795171 PMC4000654

[45] Ma Y Shimizu Y Mann MJ Jin Y Hendershot LM. Plasma cell differentiation initiates a limited ER stress response by specifically suppressing the PERK-dependent branch of the unfolded protein response. Cell Stress Chaperones. 2010;15:281–293. doi: 10.1007/s12192-009-0142-9 19898960 10.1007/s12192-009-0142-9 PMC2866998

[46] Iwakoshi NN Pypaert M Glimcher LH. The transcription factor XBP-1 is essential for the development and survival of dendritic cells. J Exp Med. 2007;204:2267–2275. doi: 10.1084/jem.20070525 17875675 10.1084/jem.20070525 PMC2118458

[47] Halliday M Radford H Sekine Y Moreno J Verity N le Quesne J Ortori CA Barrett DA Fromont C Fischer PM. Partial restoration of protein synthesis rates by the small molecule ISRIB prevents neurodegeneration without pancreatic toxicity. Cell Death Dis. 2015;6:e1672. doi: 10.1038/cddis.2015.49 25741597 10.1038/cddis.2015.49 PMC4385927

[48] Grandjean JMD Wiseman RL. Small molecule strategies to harness the unfolded protein response: where do we go from here? J Biol Chem. 2020;295:15692–15711. doi: 10.1074/jbc.REV120.010218 32887796 10.1074/jbc.REV120.010218 PMC7667976

[49] Marciniak SJ Chambers JE Ron D. Pharmacological targeting of endoplasmic reticulum stress in disease. Nat Rev Drug Discov. 2022;21:115–140. doi: 10.1038/s41573-021-00320-3 34702991 10.1038/s41573-021-00320-3

[50] Apel F Andreeva L Knackstedt LS Streeck R Frese CK Goosmann C Hopfner KP Zychlinsky A. The cytosolic DNA sensor cGAS recognizes neutrophil extracellular traps. Sci Signal. 2021;14:eaax7942. doi: 10.1126/scisignal.aax7942 33688080 10.1126/scisignal.aax7942

[51] Chen K Murao A Arif A Takizawa S Jin H Jiang J Aziz M Wang P. Inhibition of efferocytosis by extracellular CIRP-induced neutrophil extracellular traps. J Immunol. 2021;206:797–806. doi: 10.4049/jimmunol.2000091 33380498 10.4049/jimmunol.2000091 PMC7854515

[52] Shamsuzzaman S Deaton RA Salamon A Doviak H Serbulea V Milosek VM Evans MA Karnewar S Saibaba S Alencar GF. Novel mouse model of myocardial infarction, plaque rupture, and stroke shows improved survival with myeloperoxidase inhibition. Circulation. 2024;150:687–705. doi: 10.1161/CIRCULATIONAHA.123.067931 38881440 10.1161/CIRCULATIONAHA.123.067931 PMC11347105

[53] Cao J Roth S Zhang S Kopczak A Mami S Asare Y Georgakis MK Messerer D Horn A Shemer R; DEMDAS Study Group. DNA-sensing inflammasomes cause recurrent atherosclerotic stroke. Nature. 2024;633:433–441. doi: 10.1038/s41586-024-07803-4 39112714 10.1038/s41586-024-07803-4 PMC11390481

