# Supplementary material for: Macrophage DNases Limit Neutrophil Extracellular Trap–Mediated Defective Efferocytosis in Atherosclerosis
Source: Circ Res. 2025 Oct 1;137(10):1255–75. doi: 10.1161/CIRCRESAHA.125.326353 (PMC12542999; doi:10.1161/CIRCRESAHA.125.326353)
Supplement: Supplementary file 1 [file res-137-1255-s001.pdf]

Supplemental Material

Macrophage DNases Limit Neutrophil Extracellular Trap Mediated Defective Efferocytosis in Atherosclerosis

Umesh Kumar Dhawan[1], Tanwi Vartak[2], Hanna Englert[3], Stefan Russo[1], Luiz Ricardo C. Vasconcellos[4], Aarushi Singhal[1], Rahul Chakraborty[5], Karran Kiran Bhagat[1], Ciaran McDonnell[6], Mary Connolly[6], Edward Mulkern[6], Martin O'Donohoe[6], Mathias Gelderblom[7], Thomas Renne[3,8,9], Catherine Godson[2], Eoin Brennan[2], Manikandan Subramanian[1]

[1], William Harvey Research Institute, Queen Mary University of London, London, UK

[2], Diabetes Complications Research Centre, Conway Institute and School of Medicine, University College Dublin, Dublin 4, Ireland

[3], Institute of Clinical Chemistry and Laboratory Medicine, University Medical Center Hamburg-Eppendorf, Hamburg, Germany

[4], The Francis Crick Institute, London, UK

[5], CSIR-Institute of Genomics and Integrative Biology, New Delhi, India

[6], Department of Vascular Surgery, Mater Misericordiae University Hospital, Dublin, Ireland

[7], Department of Neurology, University Medical Center Hamburg-Eppendorf, Hamburg, Germany

[8], Irish Centre for Vascular Biology, School of Pharmacy and Biomolecular Sciences, Royal College of Surgeons in Ireland, Dublin, Ireland

[9], Center for Thrombosis and Hemostasis (CTH), Johannes Gutenberg University Medical Center, Mainz, Germany

Figure S1

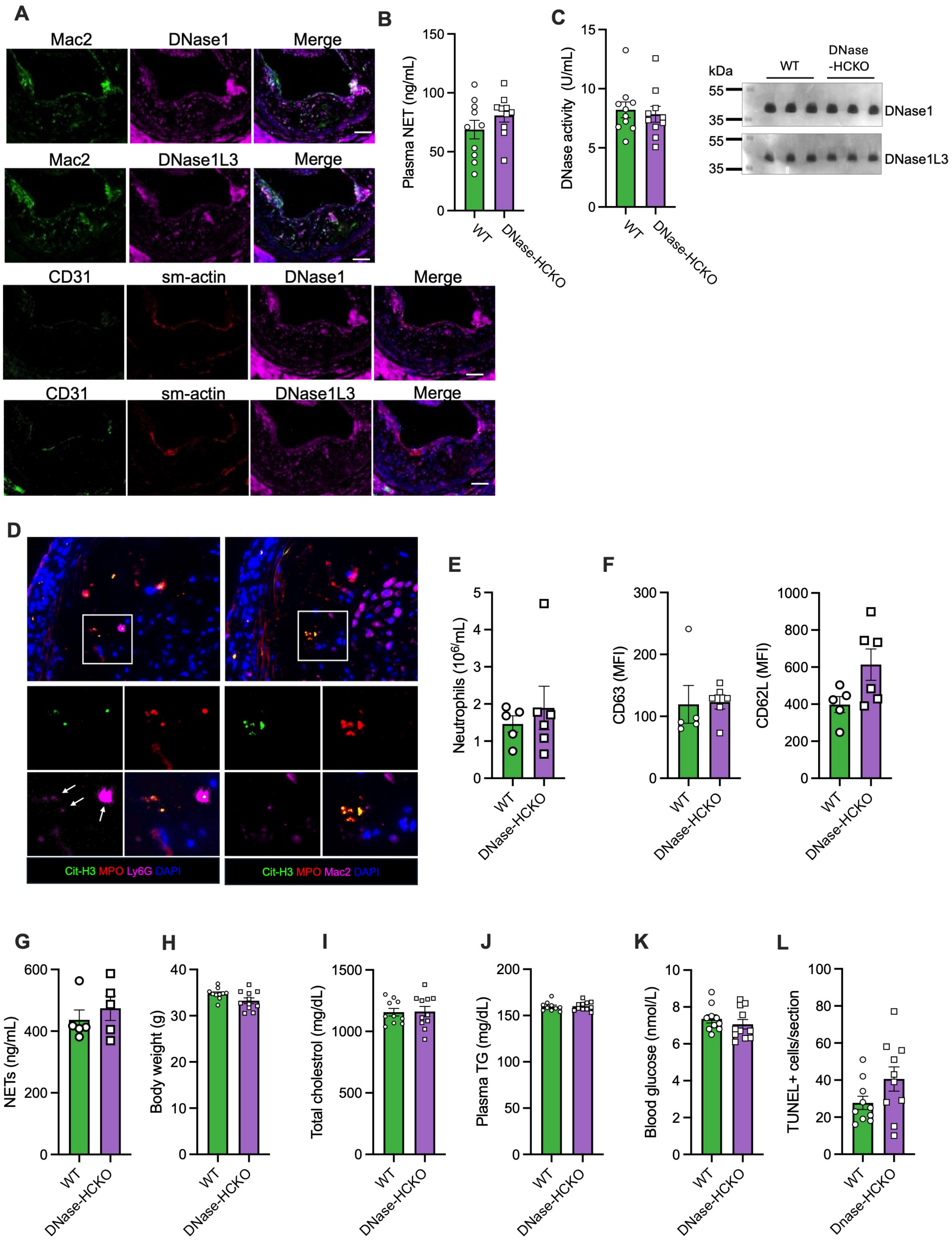

A
Mac2
DNase1
Merge
DNase1L3
CD31
sm-actin
B
Plasma NET (ng/mL)
WT
DNase-HCKO
C
DNase activity (U/mL)
kDa
55
35
DNase1L3
D
Cit-H3 MPO Ly6G DAPI
Cit-H3 MPO Mac2 DAPI
E
Neutrophils (10^6/mL)
F
CD63 (MFI)
CD62L (MFI)
G
NETs (ng/mL)
H
Body weight (g)
I
Total cholestrol (mg/dL)
J
Plasma TG (mg/dL)
K
Blood glucose (nmol/L)
L
TUNEL+ cells/section
Dnase-HCKO

Figure S1. **(A)** Representative fluorescence microscopy images of aortic root sections from 16 wk WD-fed *Ldlr*$^{-/-}$ mice immunostained with anti-Mac2, anti-sm-actin, anti-CD31, anti-DNase1, and anti-DNase1L3 antibody. **(B)** Measurement of plasma DNase activity in 16 wk WD-fed WT and DNase-HCKO *Ldlr*$^{-/-}$ mice. **(C)** Analysis of relative levels of plasma DNase1and DNase1L3 by DPZ in 16 wk WD-fed WT and DNase-HCKO *Ldlr*$^{-/-}$ mice. (**D**) Serial aortic root sections were immunostained with anti-CitH3 and anti-MPO, and either anti-Ly6G or anti-Mac2 antibodies. The colocalization of the CitH3 and MPO signal with either Ly6G or Mac2 was analyzed. The white arrows indicate colocalization of CitH3 and MPO signal with Ly6G staining. **(E)** Quantification of peripheral blood neutrophil numbers in WT and DNase-HCKO mice. (**F**) Flow-cytometry based analysis of levels of CD63 and CD62L in peripheral blood neutrophils (CD45^{+}Ly6G^{+}) in WT and DNase-HCKO mice. (**G**) Peripheral blood neutrophils isolated from WT and DNase-HCKO mice were exposed to PMA to induce NETosis. NET levels were quantified by MPO-DNA ELISA. **(H)** Body weight of WT and DNase-HCKO *Ldlr*$^{-/-}$ mice at 16 wks of WD feeding. **(I)** Quantification of total cholesterol, and **(J)** triglycerides in plasma of 16 wk WD-fed WT and DNase-HCKO *Ldlr*$^{-/-}$ mice. **(K)** Analysis of blood glucose levels in 16 wk WD-fed WT and DNase-HCKO *Ldlr*$^{-/-}$ mice. (**L**) Quantification of total number of lesional TUNEL+ cells per intimal section in 16 wk WD-fed WT and DNase-HCKO *Ldlr*$^{-/-}$ mice. n = 10 mice per group. The data are represented as mean ± SEM. Data were tested for normal distribution using Shapiro-Wilk test. P values were calculated using unpaired t-test (B, C, E-L).

Figure S2

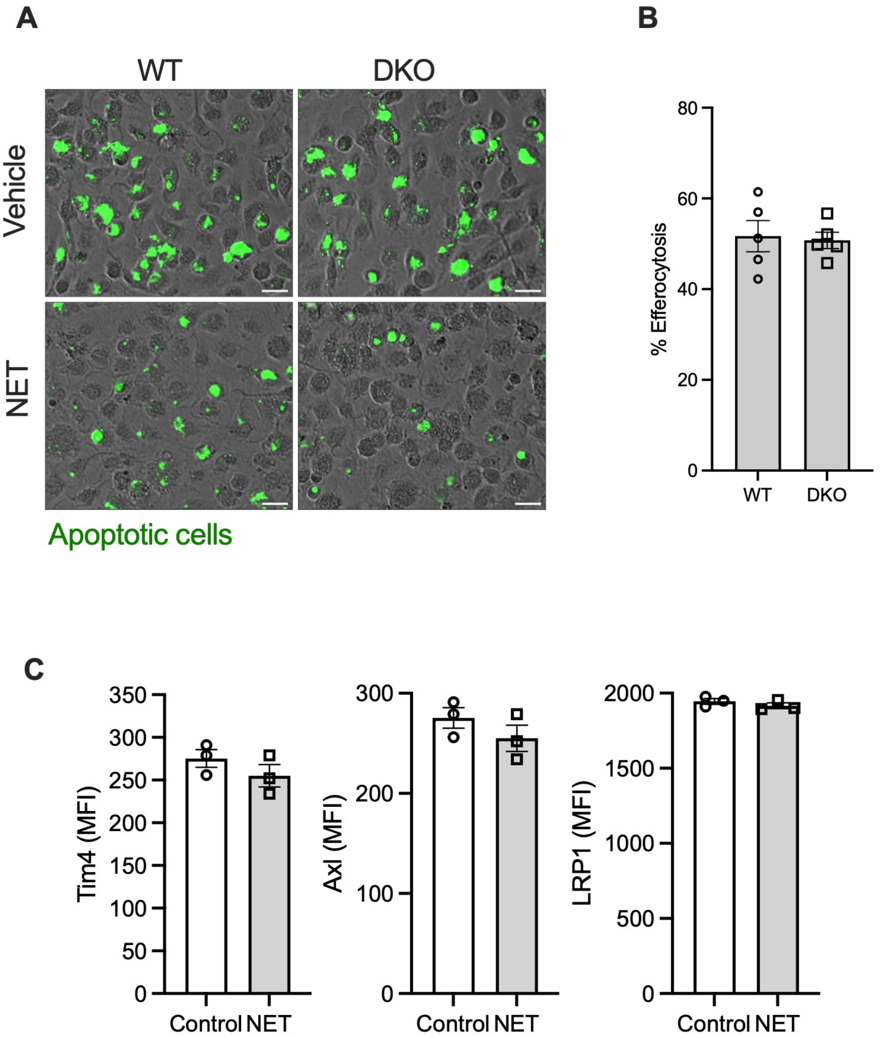

Figure S2. (A) WT and DKO BMDMs were exposed to vehicle or NETs for 2 h followed by incubation with fluorescently labelled apoptotic cells for quantification of efferocytosis efficiency. Representative fluorescence microscopy images are shown. **(B)** The bar graph shows efferocytosis quantification of WT and DKO BMDMs under homeostatic conditions. n = 5 biological replicates. **(C)** The bar graphs represent flow cytometric quantification of cell surface levels of Tim4, Axl, and LRP1, in macrophages exposed to vehicle or NETs. n = 3 biological replicates. P values were calculated using Mann-Whitney U test (B).

Figure S3

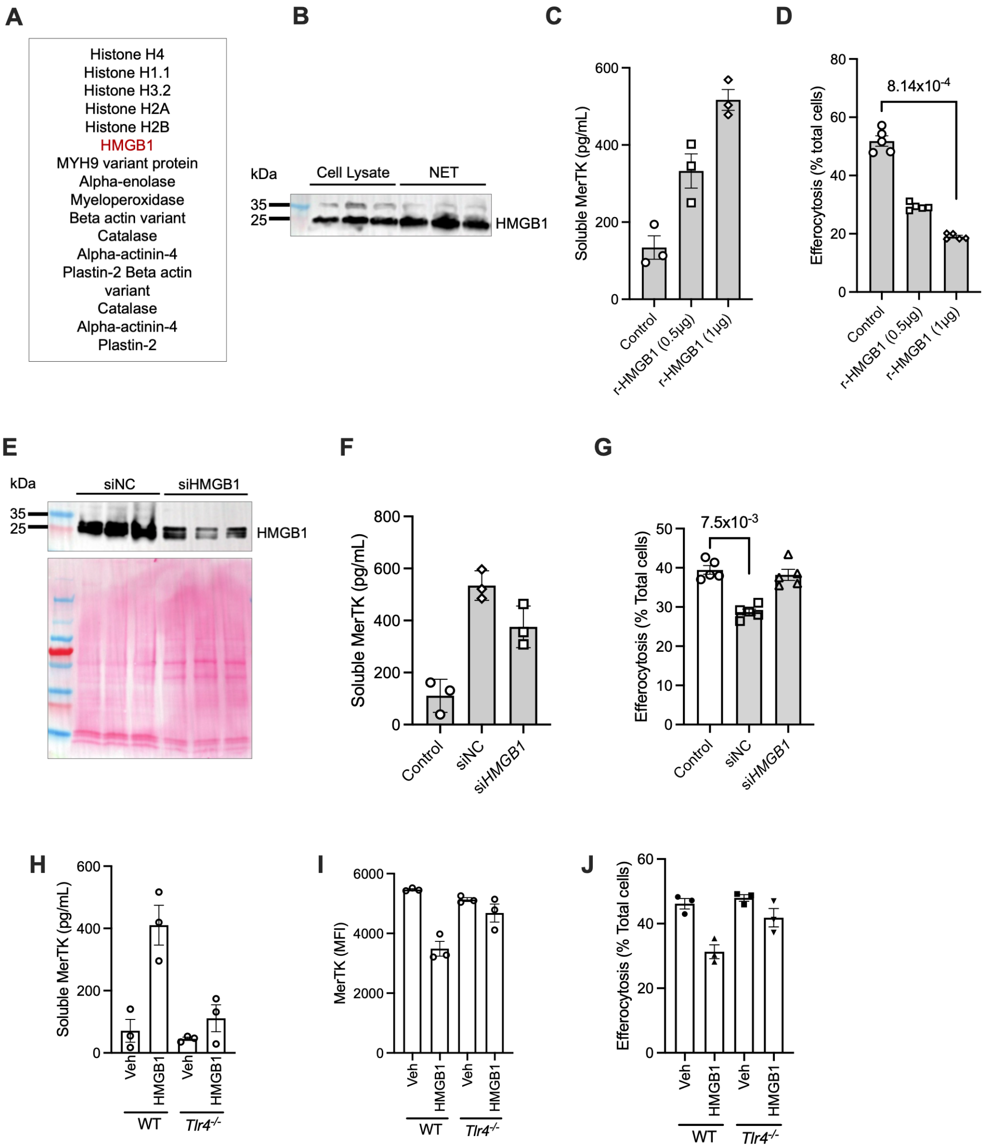

Figure S3. (A) The table shows a list of NET-associated proteins detected by mass spectrometry. **(B)** Immunoblotting for HMGB1 in ATRA-differentiated HL60 cell lysates and NETs. **(C)** BMDMs were incubated with recombinant HMGB1 for 2 h followed by measurement of soluble-Mertk in the supernatant. n = 3 biological replicates. **(D)** As above, except that HMGB1 exposed macrophages were incubated with apoptotic cells for quantification of efferocytosis efficiency. n = 4 biological replicates. **(E)** Differentiated HL-60 cells transfected with siNC or siHMGB1 were cultured for 24 h before PMA-induced NETosis. NETs were analyzed by western blot for HMGB1. Bottom panel shows Ponceau S-stained membrane. **(F-G)** Soluble Mertk levels and efferocytosis efficiency was analyzed in BMDMs incubated with NETs derived from HL60 cells transfected with siNC or siHMGB1. **(H-J)** Soluble-Mertk levels, cell Mertk levels, and efferocytosis efficiency was quantified in WT and *Tlr4*$^{-/-}$ BMDMs exposed to recombinant HMGB1(0.5 μg) for 2 h. The data are represented as mean ± SEM. P values were calculated using Kruskal-Wallis test with Dunn's multiple comparisons correction (D, G).

Figure S4

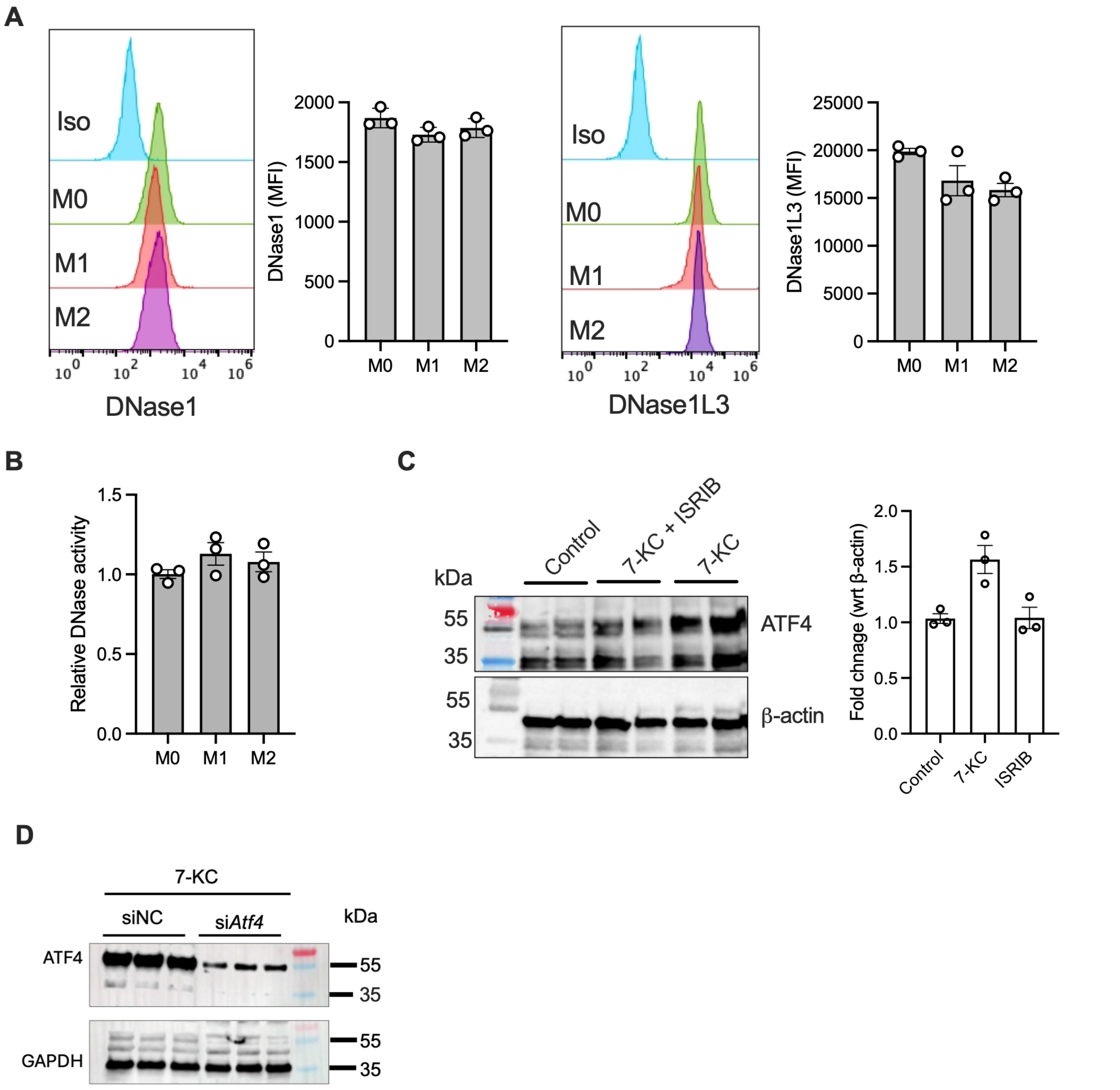

Figure S4. (**A**) BMDMs were polarized to M1 and M2 macrophages by exposure to LPS and IL4 respectively, and the intracellular levels of DNase1 and DNase1L3 were measured in fixed and permeabilized cells by flow cytometry using following immunostaining with anti-DNase1 and anti-DNase1L3 antibodies. n = 3 biological replicates. (**B**) As above, BMDMs were polarized to M1 or M2 phenotype followed by incubation with NETs for 2 h and the NET-induced DNase response was measured by SRED-based analysis of DNase activity in the cell culture supernatant. n = 3 biological replicates. **(C)** Immunobloting for ATF4 in lysates obtained from BMDMs treated with 7-KC in the absence or presence of ISRIB. **(D)** Immunobloting for ATF4 in siNC or si*Atf4* transfected BMDMs treated with 7-KC. n = 3 biological replicates.

Figure S5

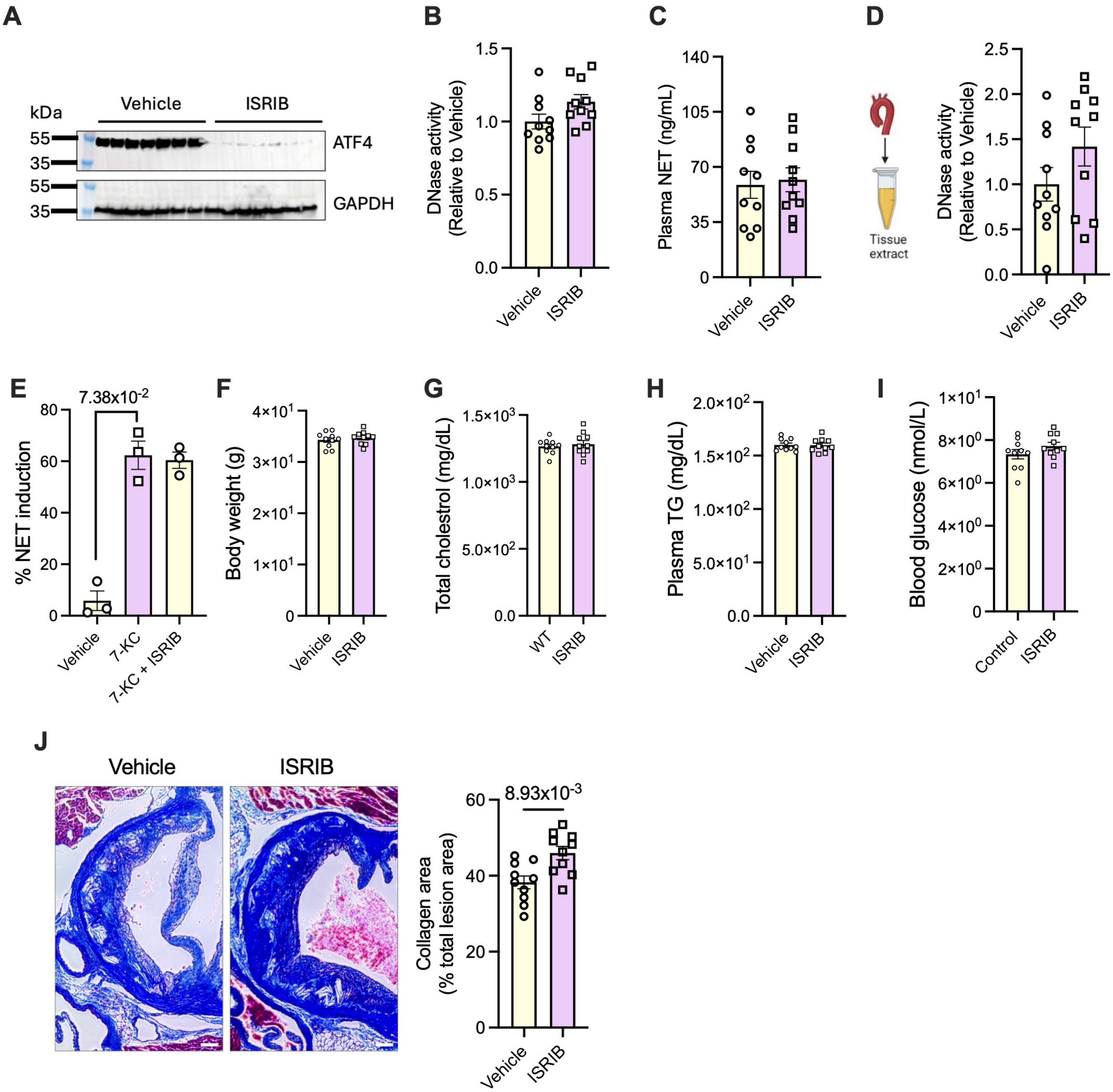

Figure S5. *Ldlr*$^{-/-}$ mice fed a WD for 16 weeks were treated with either vehicle or ISRIB for the last 4 weeks. The following parameters were quantified: **(A)** ATF4 levels in aortic tissue by western blotting; **(B)** plasma DNase activity; **(C)** Plasma level of NETs; **(D)** basal DNase activity in the aortic tissue; **(E)** Quantification of 7-KC-induced NETosis in vehicle and ISRIB-treated neutrophils; **(F)** body weight; **(G)** plasma total cholesterol; **(H)** plasma triglycerides; **(I)** blood glucose; and **(J)** Quantification of lesional collagen content by Mason's trichrome staining in aortic root sections of vehicle and ISRIB treated *Ldlr*$^{-/-}$ mice. n = 10 mice per group. The data are represented as mean ± SEM. Data were tested for normal distribution using Shapiro-Wilk test. P values were calculated using unpaired t-test (B-D, F-J) and Kruskal-Wallis test with Dunn's multiple comparisons correction (E).

<u>**Table 1**</u>

Major Resources Table

Animals

**Mouse Strain**	**Vendor or Source**	**Background Strain**	**Sex**
C57BL/6J	Charles River, UK	C57BL/6J	F/M
Ldlr$^{-/-}$	The Jackson Laboratory	C57BL/6J	F
Dnase1$^{-/-}$*Dnase1l3*$^{-/-}$	University Medical Center-Eppendorf	C57BL/6J	F
Mertk^{CR}	Columbia University Medical Center, NY	C57BL/6J	M
Tlr4$^{-/-}$	Osaka University, Japan	C57BL/6	F

Antibodies

**Target antigen**	**Vendor or Source**	**Catalog #**	**Working dilution**	**Persistent ID / URL**
F4/80	Proteintech	29414-1-AP	6 µg/ml	RRID: AB_2918300
Cit-H3	Novus Biologicals	NB500-171	10 µg/ml	RRID: AB_10001790
Anti-Actin, alpha-SmoothMuscle-Cy3(TM)	Sigma	C6198	10 µg/ml	RRID: AB_476856
DNase1	Invitrogen	PA5-52731	10 µg/ml	RRID: AB_2640724
DNase1L3	Invitrogen	PA5-30006	10 µg/ml	RRID: AB_2547480
Purified-mouse/humanMac-2 (Galectin3)	Biolegend	125402	10 µg/ml	RRID: AB_1134238
MERTK (DS5MMER)-PE	eBioscience	12-5751-82	1 µg/ml	RRID: AB_2572623
MPO	Invitrogen	PA5-16672	1:1000 - ELISA, 1:100 - IHC	RRID:AB_11006367
Mouse Axl Affinity	R&D Systems	BAF854	2.5 µg/ml	AB_2290214

Purified PAb mouse				
Monoclonal Anti-LRP1	Sigma	L2420	10 µg/ml	RRID: AB_10604109
TIM-4 (54(RMT4-54))-PE	eBioscience	12-5866-82	2 µg/ml	RRID: AB_1257163
Ly6C-BV785	Biolegend	128041	2 µg/ml	RRID: AB_2565852
Ly6G-FITC	BD biosciences	551460	5 µg/ml	RRID: AB_394207
Live/Dead TM Dead Cell Stain Kit	Thermofisher	L10119	1: 1000	https://www.thermofisher.com/order/catalog/product/L34960
CD62L-APC	Biolegend	104412	2 µg/ml	RRID: AB_313099
CD45-Pacific blue	Invitrogen	MCD4528	1:200	RRID: AB_10373710

Cultured Cells

Name	Vendor or Source	Sex (F, M, or unknown)
Human HL-60	ATCC	F
L-929	ATCC	M

Primer Sequence

Primer Name	Primer sequence 5’-3’
Mouse: TNF α- Forward	CCCTCACACTCAGATCATCTTCT
Mouse: TNF α- Reverse	GCTACGACGTGGGCTACAG
Mouse: IL1 β - Forward	GCAACTGTTCCTGAACTCAACT
Mouse: IL1 β - Reverse	ATCTTTTGGGGTCCGTCAACT
Mouse: 18 S - Forward	GTAACCCGTTGAACCCCATT
Mouse: 18 S - Reverse	CCATCCAATCGGTAGTAGCG
Mouse: IL6 -Forward	TAGTCCTTCCTACCCCAATTTCC
Mouse: IL6 -Reverse	TTGGTCCTTAGCCACTCCTTC

ARRIVE GUIDELINES

The ARRIVE guidelines (https://arriveguidelines.org/) are a checklist of recommendations to improve the reporting of research involving animals. Key elements of the study design should be included below to better enable readers to scrutinize the research adequately, evaluate its methodological rigor, and reproduce the methods or findings.

Study Design

Groups	Sex	Age	Number (prior to experiment)	Number (after termination)	Littermates (Yes/No)	Other description
2 – 3 groups per experiment	M/F as indicated in the figure legend.	8-12 weeks	N/A	Indicated in the figure legends.	No	N/A

Sample Size:

Sample sizes were determined using power calculations based on variability data from previous or pilot experiments. The calculations were designed to detect a 30% difference with 80% power at a significance level of $\alpha = 0.05$.

Inclusion Criteria

All mice were included in the study.

Exclusion Criteria

None.

Randomization

Mice were randomly assigned to different groups.

Blinding

Data analysis was conducted in a blinded manner.
