# Supplementary material for: Macrophage DNases Limit Neutrophil Extracellular Trap–Mediated Defective Efferocytosis in Atherosclerosis
Source: Circ Res. 2025 Oct 1;137(10):1255–75. doi: 10.1161/CIRCRESAHA.125.326353 (PMC12542999; doi:10.1161/CIRCRESAHA.125.326353)
Supplement: Supplementary file 2 [file res-137-1255-s002.pdf]

Figure S1C

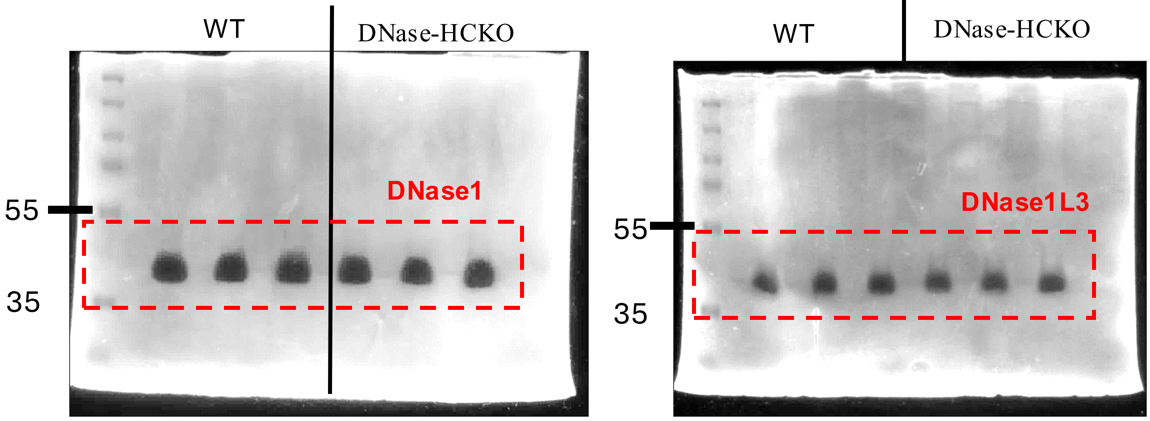

Figure S3B

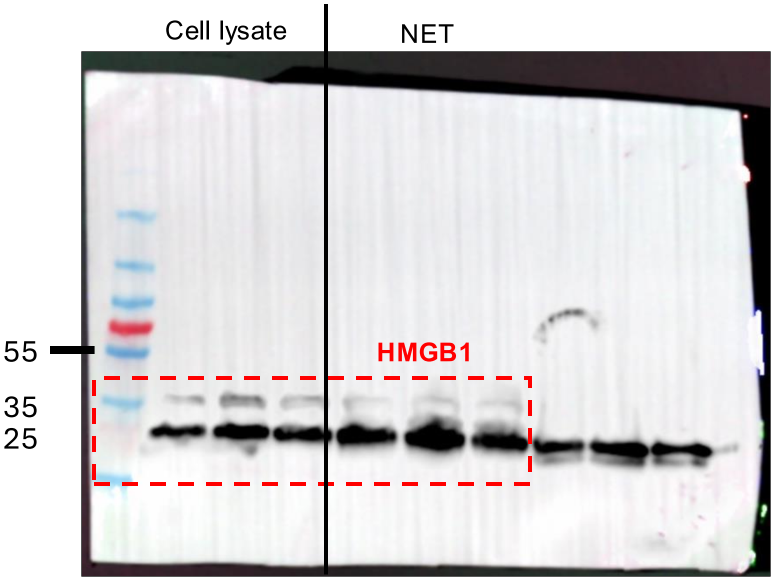

Figure S3E

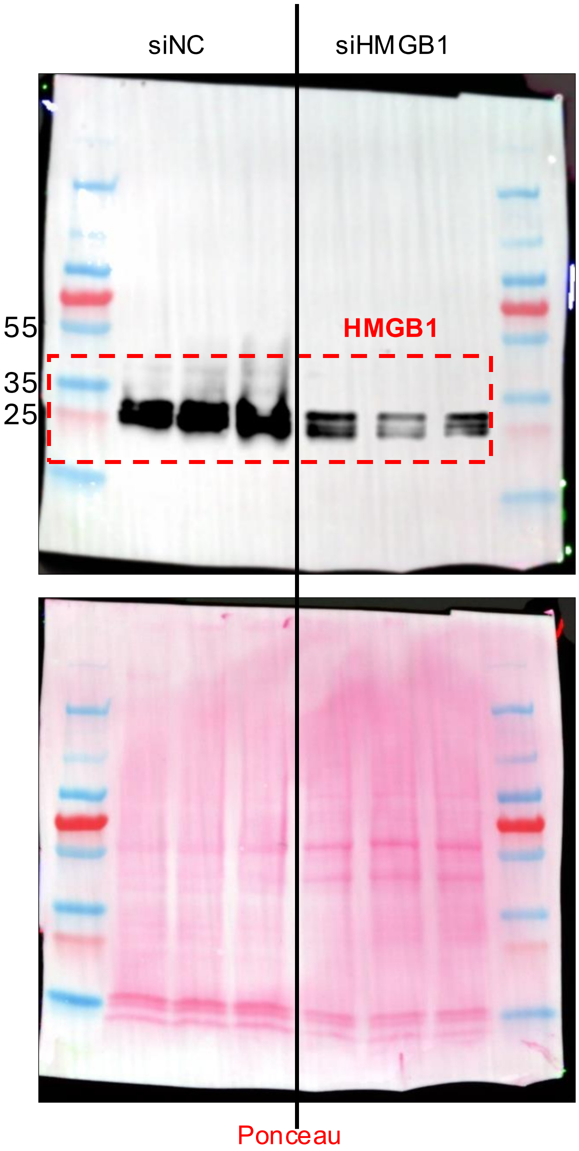

Figure 6F

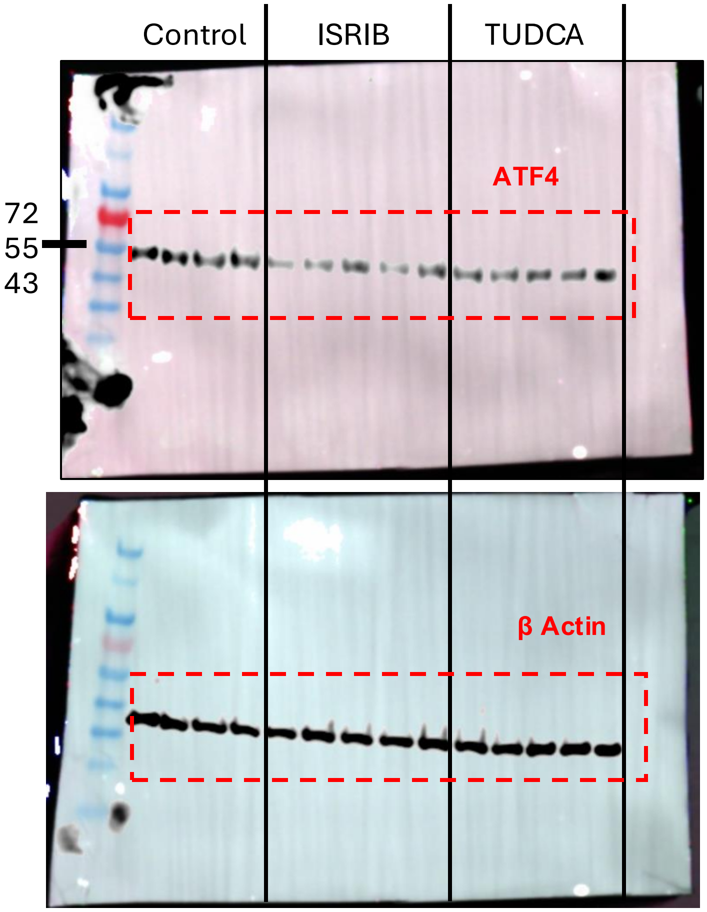

Figure 6B

Figure S4C Data for quantification

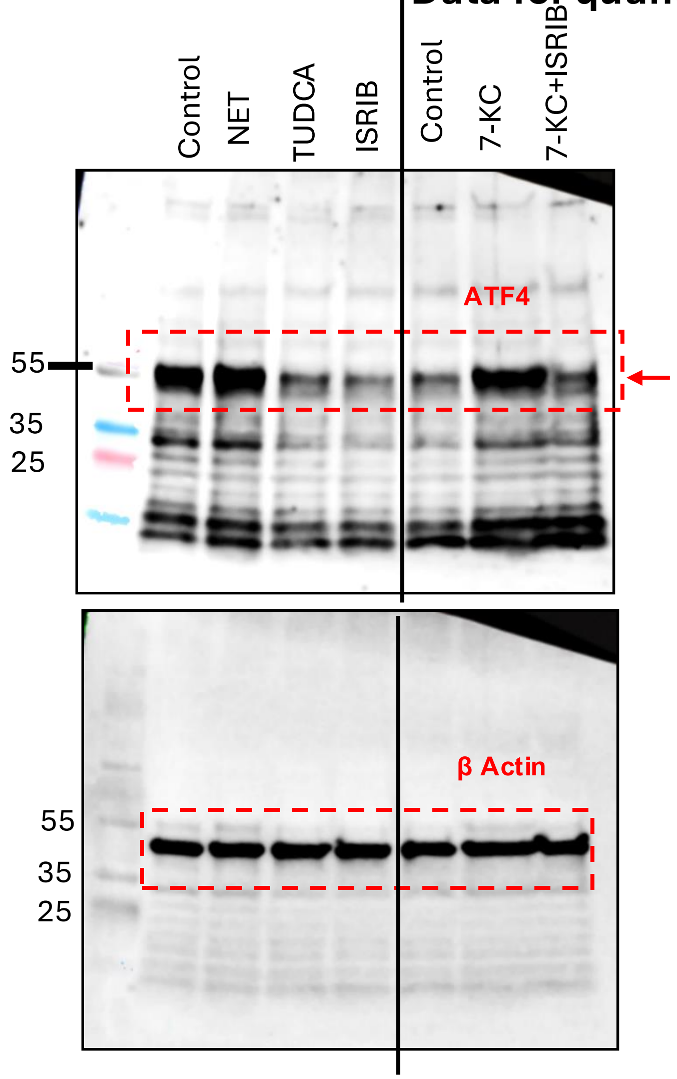

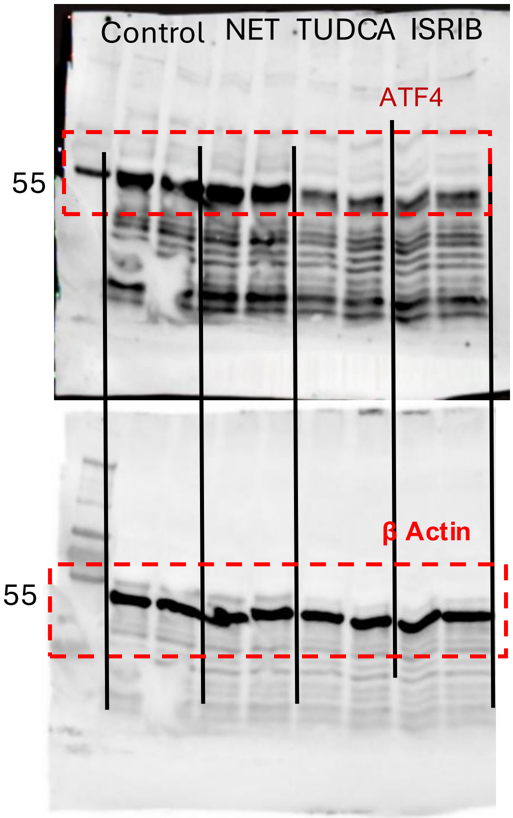

Figure S4C

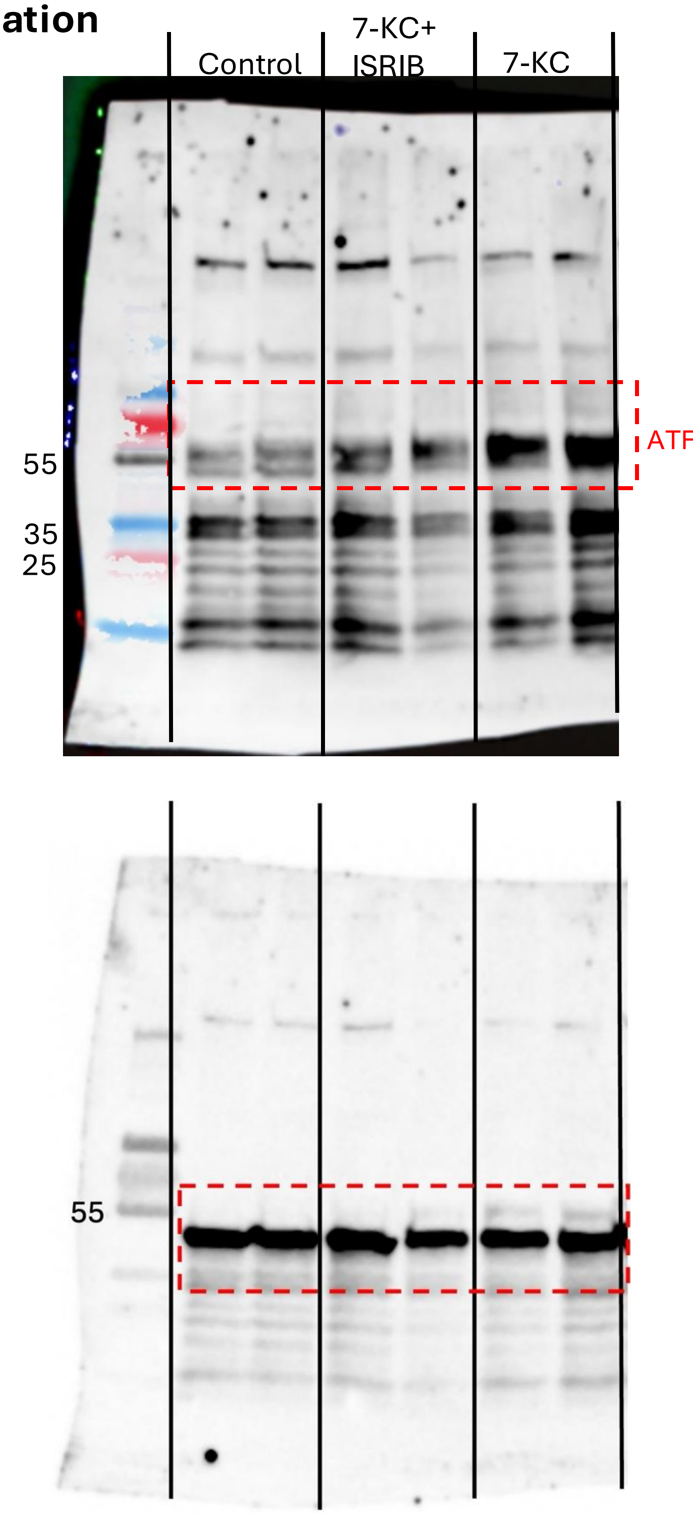

Figure S4D

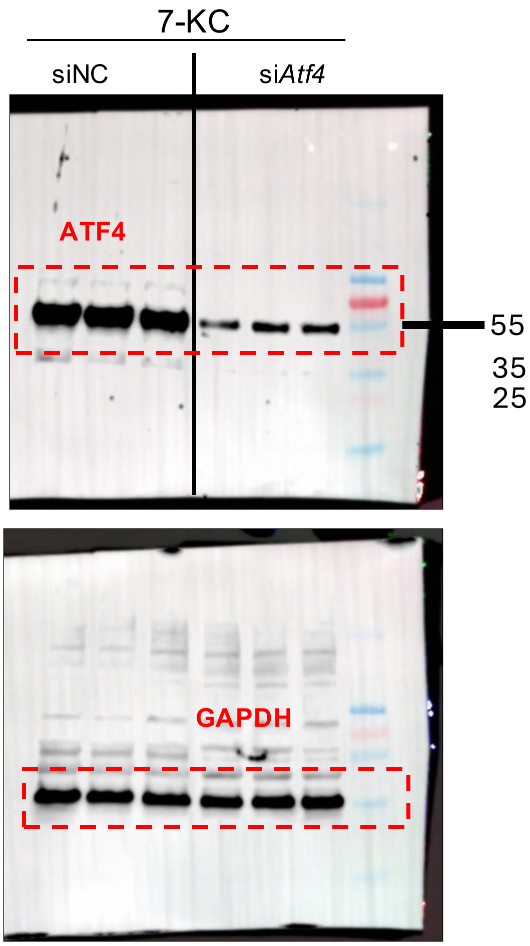

Figure S5A

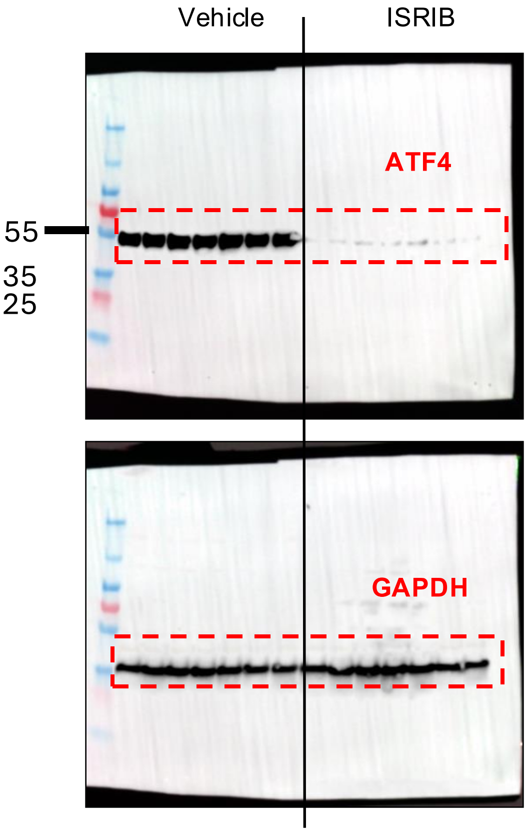

Fig. 6B

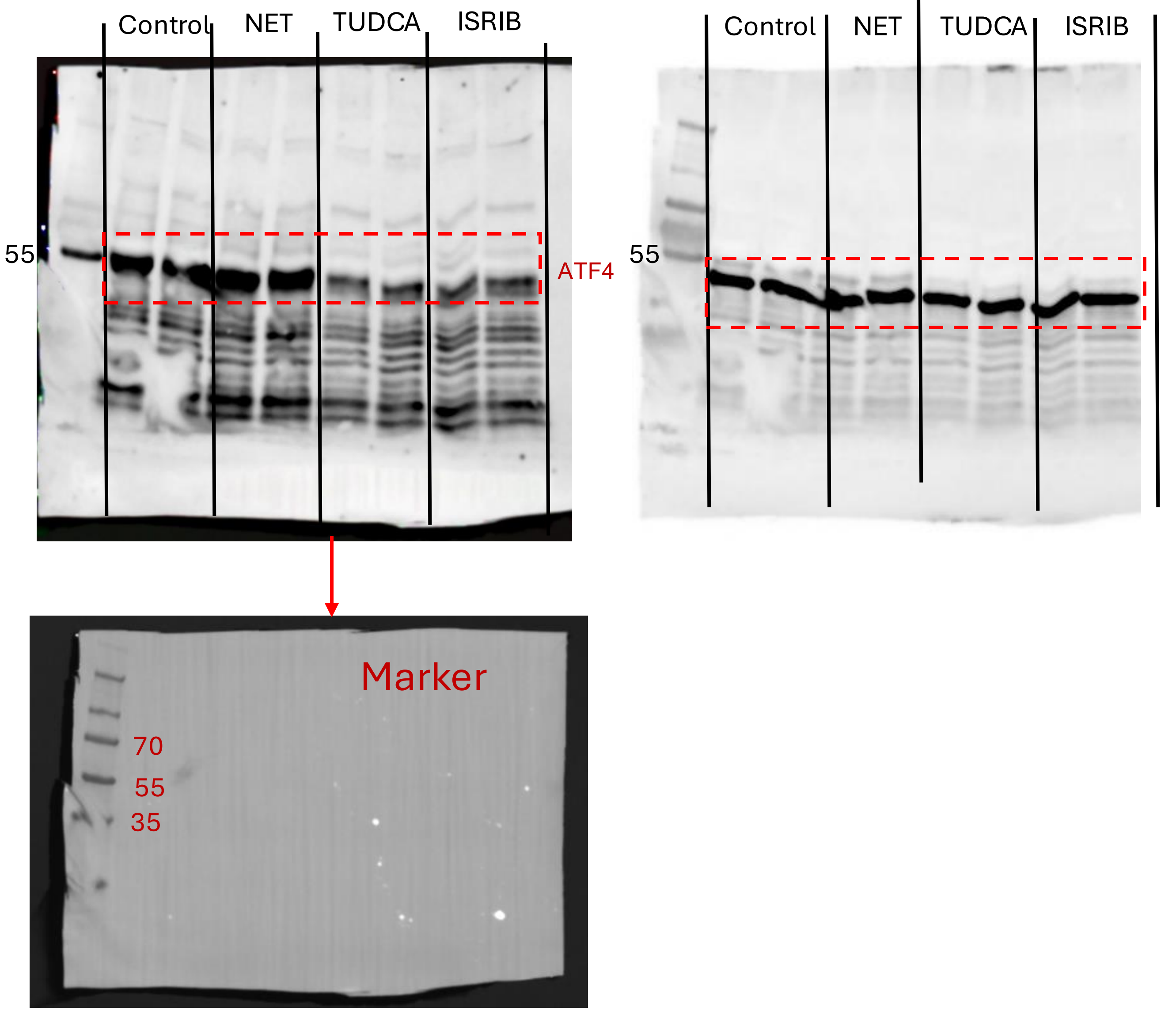
Control
NET
TUDCA
ISRIB
55
ATF4
Control
NET
TUDCA
ISRIB
55
Marker
70
55
35
